# Three doses of BNT162b2 COVID-19 mRNA vaccine establish long-lasting CD8^+^ T cell immunity in CLL and MDS patients

**DOI:** 10.3389/fimmu.2022.1035344

**Published:** 2023-01-10

**Authors:** Susana Patricia Amaya Hernandez, Ditte Stampe Hersby, Kamilla Kjærgaard Munk, Tripti Tamhane, Darya Trubach, Maria Tagliamonte, Luigi Buonaguro, Anne Ortved Gang, Sine Reker Hadrup, Sunil Kumar Saini

**Affiliations:** ^1^ Department of Health Technology, Section of Experimental and Translational Immunology, Technical University of Denmark, Kongens Lyngby, Denmark; ^2^ Department of Hematology, Copenhagen University Hospital, Rigshospitalet, Copenhagen, Denmark; ^3^ Innovative Immunological Models Unit, National Cancer Institute Pascale Foundation – IRCCS, Napoli, Italy

**Keywords:** SARS – CoV – 2, T-cell immunity, mRNA vaccine against SARS-CoV-2, Hematological cancer, CD8+ T cell, T cell memory

## Abstract

Patients with hematological malignancies are prioritized for COVID-19 vaccine due to their high risk for severe SARS-CoV-2 infection-related disease and mortality. To understand T cell immunity, its long-term persistence, and its correlation with antibody response, we evaluated the BNT162b2 COVID-19 mRNA vaccine-specific immune response in chronic lymphocytic leukemia (CLL) and myeloid dysplastic syndrome (MDS) patients. Longitudinal analysis of CD8^+^ T cells using DNA-barcoded peptide-MHC multimers covering the full SARS-CoV-2 Spike-protein (415 peptides) showed vaccine-specific T cell activation and persistence of memory T cells up to six months post-vaccination. Surprisingly, a higher frequency of vaccine-induced antigen-specific CD8^+^ T cells was observed in the patient group compared to a healthy donor group. Furthermore, and importantly, immunization with the second booster dose significantly increased the frequency of antigen-specific CD8^+^ T cells as well as the total number of T cell specificities. Altogether 59 BNT162b2 mRNA vaccine-derived immunogenic responses were identified, of which 23 established long-term CD8^+^ T cell memory response with a strong immunodominance for NYNYLYRLF (HLA-A24:02) and YLQPRTFLL (HLA-A02:01) epitopes. In summary, we mapped the vaccine-induced antigen-specific CD8^+^ T cells and showed a booster-specific activation and enrichment of memory T cells that could be important for long-term disease protection in this patient group.

## Introduction

Patients with chronic lymphocytic leukemia (CLL) and Myelodysplastic syndrome (MDS) are generally faced with immune defects and deficiencies, related to their primary disease and as a result of their cancer treatments, that may impact the immune cells’ ability to evade infections (1–3). Due to the increased risk for severe SARS-CoV-2 infection and mortality (4–6), patients with hematological malignancies (HM) were prioritized for a vaccine-mediated protective immunity, initially with a two-dose COVID-19 vaccine and subsequently with an additional booster dose. However, we have limited knowledge on vaccine-induced T cell immunity, which could be critical for proper assessment of vaccine-mediated long-term protection (7, 8), especially since inconsistent and impaired vaccine-induced humoral responses have been reported in HM patients (9–13). Here, we report antigen-specific CD8^+^ T cell response induced by BNT162b2 COVID-19 mRNA vaccine up to six months post-vaccination, and provide a detailed characterization of T cell immunodominance, immunological memory, and impact of the booster immunization in CLL and MDS patients.

## Materials and methods

### Clinical samples

The patient cohort consists of 28 hematological patients: 23 with chronic lymphocytic leukemia (CLL) and 5 with myelodysplastic syndrome (MDS) (median age=71.8 years). The patients were screened and selected by attending physicians with the inclusion criteria of age above 18 years, a diagnosis of CLL or MDS, individuals who were prioritized for vaccination and who did not receive ongoing treatment affecting the immune system, and with no prior COVID-19 infection. Ethical clearance for collection of samples was acquired from the Committee on Health Research Ethics in the Capital Region of Denmark, and all patients gave their informed written consent for inclusion in the study. Patients received either two doses of the BNT162b2 COVID-19 mRNA vaccine (no booster; n = 11) or two vaccine doses and a booster (booster; n = 17), and blood samples were collected at four time points: prior to vaccination (Pre-vac), 7 to 10 days after first vaccine dose (TP1), 2 months after first vaccination dose (TP2), and 6 months after first vaccine dose (TP3) (**Supplementary Table S1**). No samples were collected for PTVACC-06-01, -10-01, -10-02, and -13-03, excluded from analysis. Samples from healthy donors (n=19; median age= 42.2 years; **Supplementary Table S2**) who received two doses of the BNT162b2 mRNA vaccine were collected 2 months after the first vaccine dose and obtained from the National Cancer Institute Foundation Pascale, Napoli, Italy. The study was approved by the institutional ethics committee with the inclusion criteria of age between 18 and 70 years.

### PBMC isolation and HLA genotyping

Peripheral blood mononuclear cells (PBMC) were isolated from blood samples by density gradient centrifugation using Leucosep tubes (Greiner Bio-One 227288) with Lymphoprep media (StemCell Technologies 07861) and thereafter cryopreserved in Fetal Bovine Serum (FCS; Gibco 10500-064) with 10% DMSO until analysis. PBMC samples from HM patients were genotyped for HLA-A, B, and C loci (DKMS Life Science Lab GmbH, Germany; next-generation sequencing) (**Supplementary Table S3**). Healthy donor samples were genotyped for HLA-A loci (Laboratorio di Istoconpatibilità, UOSD Criopreservazione e BaSCO, AORN Santobono-Pausilipon, Napoli, Italy) (**Supplementary Table S4**).

### Peptides

All peptides used in the analysis were custom synthesized by Pepscan (Pepscan Presto BV, Lelystad, Netherlands; **Supplementary Tables S5**, **S6**), dissolved to 10 mM dimethyl sulfoxide (DMSO; Sigma Aldrich D2650), and stored at -20°C until use.

### MHC class I monomer production

MHC class I monomers was produced as previously described (14, 15). Briefly, the MHC heavy chain of the selected HLA types and human ß2-microglobulin (hß2m) light chain were separately produced as inclusion bodies in *Escherichia coli* strain BL21(DE3)pLysS (Novagen 69451) using pET series expression plasmids. Solubilized heavy chain and hβ2m proteins were combined and folded *in vitro* to form functionally active HLA monomers using UV-sensitive ligands (15). Empty HLA-A02:01 and A24:02 molecules were produced as described previously (16). Monomers were biotinylated with BirA biotin-protein ligase standard reaction kit (Avidity LLC), and purified using size exclusion chromatography (SEC-HPLC).

### Staining of antigen-specific T cells with DNA-barcoded pMHC multimers

DNA-barcoded multimer libraries for SARS-CoV-2 Spike- and CEF-derived peptides were generated as previously described by Bentzen et al. (17). Briefly, individual peptide–MHC (pMHC) complexes were generated by incubating each peptide with their respective MHC molecules using direct peptide loading (16) for HLA-A02:01 and A24:02 or by UV-mediated peptide exchange (15) for the other HLAs. The pMHC monomers were then coupled to an allophycocyanin (APC)-, for SARS-CoV-2 Spike peptides, or a phycoerythrin (PE)-, for CEF peptides, conjugated dextran attached with unique DNA barcodes. DNA-barcoded multimers were then used for the detection of pMHC-specific T cells.

PBMC from both cohorts were thawed in RPMI (Gibco 72400021) + 10% fetal bovine serum (FCS; Gibco, 10500064) + 100 µg/ml DNAse I (StemCell Technologies 07470) + 5mM MgCl_2_ and washed twice in RPMI + 10% FCS. For CLL patient samples, CD3^+^ T cells were isolated from PBMCs using the EasySep Human T-cell isolation kit as per the manufacturer’s instructions (StemCell Technologies 17951). For MDS patients and healthy cohort samples, PBMCs were used for T cell staining without isolation. Cells from all samples were then washed once in barcode cytometry buffer (BCB; PBS + 0.5% BSA + 100 μg/mL herring DNA + 2 mM EDTA).

Patient and healthy donor PBMC samples were incubated with HLA-matching SARS-CoV-2 Spike and CEF DNA-barcoded pMHC multimers for 15 min at 37°C, followed by incubation at 4 ˚C for 30 min with a phenotype antibody panel (**Supplementary Table S7**). Healthy donor PBMCs were also incubated with all the HLA-B-specific peptides of SARS-CoV-2 and CEF library. Cells were washed twice with BCB, fixed in 1% paraformaldehyde (PFA), washed twice more, and resuspended in BCB. Cells were then acquired on flow cytometer (AriaFusion, BD Biosciences) and pMHC multimer binding CD8^+^ T cells were sorted (**Supplementary Figure S1**). Sorted cells were centrifuged for 10 min at 5000 × g, and the cell pellet stored at -20°C.

### DNA-barcode sequence analysis

DNA barcodes from the isolated cells as well as from an aliquot of the multimer pool (10,000x final dilution in the PCR reaction; used as a baseline) were PCR amplified using the Taq PCR Master Mix Kit (Qiagen, 201443). Products were purified using the QIAquick PCR Purification kit (Qiagen 28104) and sequenced at PrimBio (USA) using an Ion Torrent PGM 318, or an Ion S5 530 chip (Life Technologies).

DNA barcode sequencing data were processed using the Barracoda software package (17) (https://services.healthtech.dtu.dk/service.php?Barracoda-1.8). This software calculates the number of reads and clonally reduced reads for each pMHC-associated DNA barcode, the fold change (FC) in read counts mapped to a given sample relative to the mean read counts mapped to triplicate baseline samples, the p-values, and the false-discovery rates (FDRs) as described by (17). DNA barcodes with FDR < 0.1% (corresponding to p < 0.001) and Log_2_FC >2 over the baseline values for the total pMHC library were considered significant and to be true T cell responses.

The T cell frequency for each significantly enriched barcode was calculated from the percentage read count of the barcode out of the percentage of CD8^+^ multimer^+^ T cells. A non-HLA-matching and unvaccinated healthy donor was included as a negative control and peptides recognized in this sample were subtracted from the data set to exclude potential non-specific pMHC binding to T cells.

### T cell staining with fluorescently labeled pMHC tetramers

pMHC complexes with a T cell response detected using the DNA-barcode labeled multimers were selected to generate combinatorial fluorescently labeled pMHC tetramers as previously described (18, 19). Single-fluorochrome pMHC tetramers were produced by conjugating individual pMHC complexes, generated as described above, to a library of fluorophore-labeled streptavidin (SA) molecules consisting of PE-SA (Biolegend 405204), PE-CF594-SA (BD Biosciences 562284), APC-SA (Biolegend 405207) BUV395-SA (BD Biosciences 564176), BV421-SA (BD Biosciences 563259), BV737 (BD Biosciences 564293). Each pMHC specificity was multimerized on two different SA conjugated fluorochromes for combinatorial T cell staining. PBMCs from HD and HM patient cohorts were expanded *in vitro* for 14 days using a single peptide (1 µM) and cytokines IL-2 (100 IU/mL) and IL-15 (25 IU/mL) in X-vivo media (Lonza BE02-060Q) with 5% human serum (HS; Gibco 1027-106). Expanded cells were stained to detect SARS-CoV-2 or CEF specific-T cells using pMHC tetramers or to measure intracellular cytokines for T-cell functional analysis. Primary and expanded PBMCs from both the cohorts were incubated with 1 μL of pooled pMHC tetramers per specificity together with dasatinib, followed by staining with an antibody solution mix (**Supplementary Table S7**). Cells were acquired on a LSRFortessa flow cytometer (BD Biosciences).

### T cell functional analysis by intracellular cytokine staining

The functional capacity of T cells was measured using intracellular cytokines IFN-γ and TNF-α upon stimulation with specific peptides. Expanded PBMCs, generated as described above, from two healthy donors and two HM patients were incubated in X-vivo + 5% HS + protein transport inhibitor (GolgiPlug; BD Biosciences 555029; final dilution 1/1000) and stimulated with 1 µM of SARS-CoV-2 Spike single epitope for 8 hours at 37°C, 5% CO_2_. Cells incubated with Leukocyte Activation Cocktail (BD 550583; final dilution 1/500) were used as a positive control, and cells incubated with DMSO (final dilution 1/10,000) were used as a negative control for each donor sample. Cells were stained with the surface marker antibodies listed in **Supplementary Table S7**, followed by fixation (fixation buffer; eBioscience 00-8222-49) and permeabilization (permeabilization buffer; eBioscience 00-8333-56), and finally cells were stained with the intracellular antibodies (**Supplementary Table S7**). CD8^+^ T cells producing intracellular cytokines were acquired on a LSRFortessa flow cytometer (BD Bioscience).

### Flow cytometry analysis

All flow cytometry data were analyzed using FlowJo data analysis software (version 10.8.1; FlowJo LLC). For phenotype analysis pMHC multimer positive CD8+ T-cells were analyzed according to the gating strategy shown in the **Supplementary Figure S1**. For Uniform Manifold Approximation and Projection (UMAP) analysis of SARS-CoV-2 Spike multimers-specific T cells, FCS files of samples were concatenated at the APC-positive population gate (250 events per sample), and visualized using UMAP analysis (Version 3.1 (20)) based on the markers CD38, CD39, CD69, CD137, HLA-DR, PD-1, CCR7, CD45RA, and CD27. For antigen-specific T cell identification using combinatorial pMHC tetramer staining, we gated on single, live, CD8^+^ and FITC^-^ (dump channel) lymphocytes and selected cells positive in two tetramer colors and negative in the remaining colors (**Supplementary Figure S7A**) (19). For functional evaluation, we gated on single, live, CD8^+^ lymphocytes and calculated the frequency (%) of CD8^+^ T cells double or single positive for the analyzed cytokines (**Supplementary Figure S8**).

### Multiplex immunoassay for antibody detection

The multiplex bead-binding assays Milliplex SARS-CoV-2 Antigen Panels (Millipore HC19SERM1-85K, HC19SERG1-85K and HC19SERA1-85K kits) were used to test immunoglobulin antibody levels (IgM, IgG, and IgA) against SARS-CoV-2 Spike protein subunits S1 and S2, the Spike receptor-binding domain (RBD), and nucleocapsid (N) protein. Plasma collected during PBMC isolation was centrifuged for 10 minutes at 1000 x g, diluted at 1:100 in the Assay Buffer provided in the kits, and processed as per the manufacturer’s instructions. The four control beads are combined with the four antigen-immobilized beads (S1, S2, RBD and N) and added to each sample followed by the addition of PE-anti Human IgM, IgG or IgA Conjugate. The Assay Buffer was used to measure the background non-specific binding and the performance of the assay reagents was monitored using assay-specific negative and positive controls provided by the manufacturer. All samples and controls were analyzed in single measurements. Median fluorescence intensities (MFI) were measured using a Bio-Plex MAGPIX Multiplex Reader (Bio-Rad Laboratories) in combination with xPotent software version 4.2 (Luminex Corporation). The measured MFI was multiplied by the dilution factor and corrected by subtracting background MFI.

### Multiplex immunoassay for T cell functional analysis

PBMCs (1 × 10^6^ cells) were rested in X-vivo media (Lonza BE02-060Q) with 5% HS (Gibco 1027-106) and stimulated with 1 µM of individual SARS-CoV-2 Spike epitopes or DMSO (concentration matched negative control) for 24 hours at 37°C followed by collection of 25 µL of cell supernatant to quantify the levels of TNFα and IFN-γ using MILLIPLEX^®^ Human Cytokine/Chemokine/Growth Factor Panel A bead-based multiplex assay kit (Millipore HCYTA-60K-05). Samples were processed as per manufacturer’s instructions. Two TNFα assay controls (expected ranges control 1: 94-196 pg/mL, control 2: 489-1016 pg/mL) and two IFN-γ assay controls (expected ranges control 1: 119-246 pg/mL, control 2: 574-1192 pg/mL) were included to monitor the assay performance. A seven-point standard curve was generated for each of the cytokines, standard samples were prepared by serial dilution (1:1) of TNFα (range 0-100,000 pg/mL) and IFN-γ (range 0-20,000 pg/mL) standards. Kit’s assay buffer was used to measure the background as well as the 0 pg/mL standards. Samples, controls and standards were assayed in single measurements. Data was acquired and analyzed using a Bio-Plex MAGPIX Multiplex Reader (Bio-Rad Laboratories) in combination with xPotent software version 4.2 (Luminex Corporation). Cytokine levels measured as MFI were converted to pg/mL concentration using the standard curves. Cytokine concentrations were then corrected by subtracting the DMSO negative control concentrations for each sample.

### Data processing and statistical analysis

**Figure 1:**
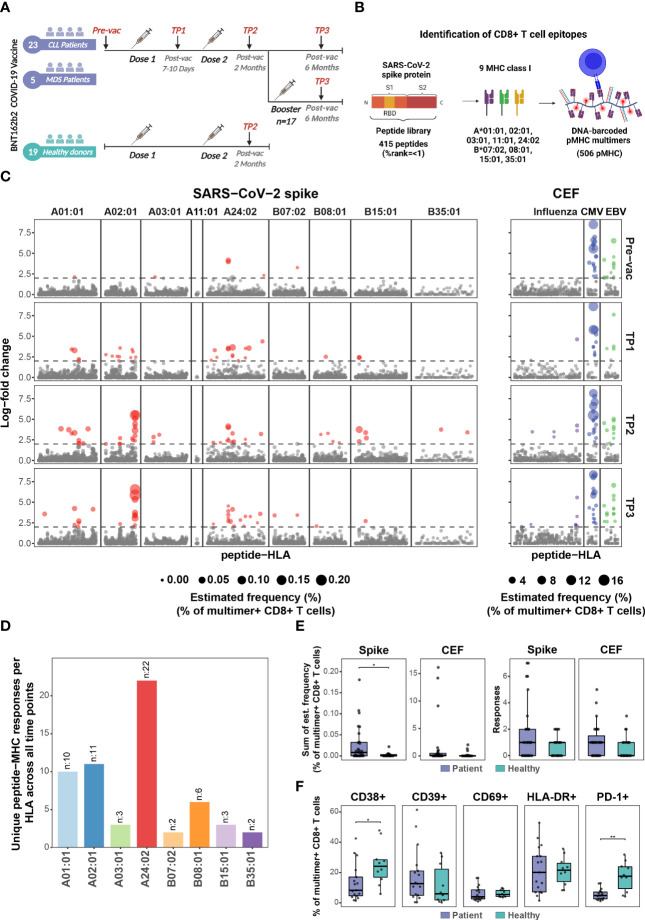
BNT162b2 COVID-19 mRNA vaccine drives early and persistent antigen-specific CD8^+^ T cells activation in CLL and MDS patients. **(A)** Sample collection timeline for patients with hematological malignancies (CLL, n=23 individuals; MDS, n=5 individuals) and healthy donors’ cohort (n=19 individuals). All donors received either two or three (booster) doses of the BNT162b2 mRNA vaccine. **(B)** Representation of the peptide library and the DNA-barcoded peptide-MHC (pMHC) multimers used to analyze T cell recognition towards the SARS-CoV-2 Spike protein-derived HLA-binding peptides. **(C)** Summary of CD8^+^ T cell recognition to SARS-CoV-2 Spike- and CEF- derived peptides in the HM patients at four time points: prior to vaccination (Pre-vac, n=28), 7 to 10 days after first vaccine dose (TP1, n=26), 2 months after first vaccination dose (TP2, n=27), and 6 months after first vaccine dose (TP3, n=27). Responses were identified based on the enrichment of DNA barcodes associated with each of the tested peptide specificities (LogFc ≥ 2 and p < 0.001, Barracoda). Each dot represents one peptide-HLA combination per patient. The size of each significant response (colored dotes) is proportional to the estimated frequency (%) calculated from the percentage read count of the associated barcode out of the percentage of CD8^+^ multimer^+^ T cells. SARS-CoV-2 Spike-specific T cell responses were segregated based on their HLA. **(D)** The bar plot summarizes the number of unique HLA-specific SARS-CoV-2 Spike epitopes identified in the HM patients across all the time points analyzed. **(E)** Box plots comparing the number of significant SARS-CoV-2 Spike- and CEF- derived peptides responses (right) and the sum of their estimated frequencies (left) for each individual in the HM patients (n=27) and the healthy donors (n=19) at TP2. Each dot represents one sample. Mann-Whitney test, Spike patients vs. healthy (p = 0.03). **(F)** Box plots show the percentage of SARS-CoV-2 Spike pMHC multimer^+^ CD8^+^ T cells expressing the indicated surface markers to compare the phenotype of the HM patients (n=27) and the healthy donors at TP2 (n=19). Mann-Whitney test, patients vs. healthy CD38^+^ (p=0.02) and patients vs. healthy PD-1^+^ (p = 0.002). * p ≤ 0.05, ** p ≤ 0.01.

**Figure 2:**
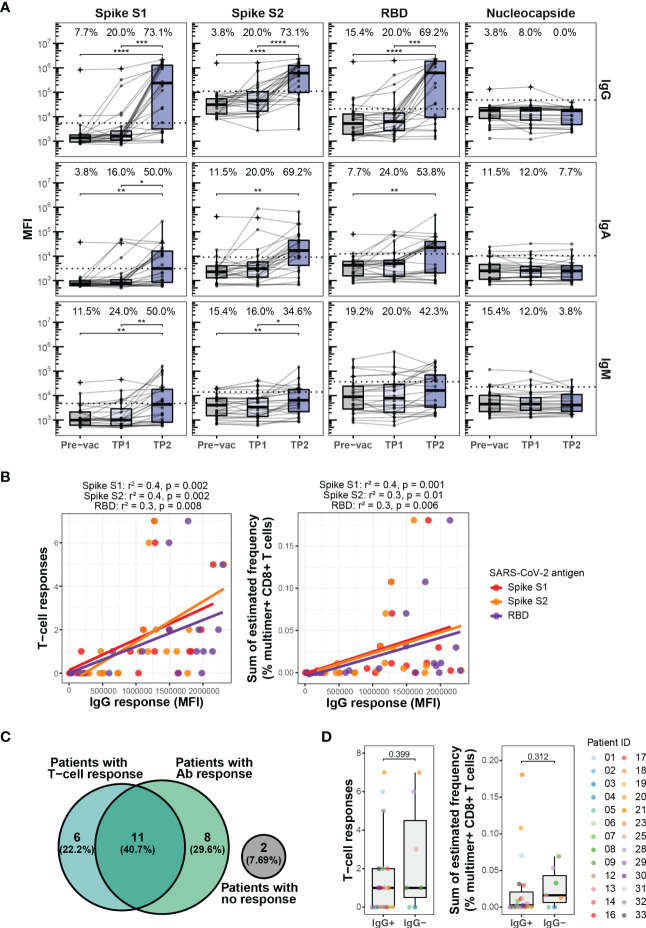
Antibody response to BNT162b2 mRNA vaccination. **(A)** Levels of IgG, IgA, and IgM antibodies against SARS-CoV-2 Spike protein subunits S1 and S2, the Spike receptor-binding domain (RBD), and nucleocapsid (N) protein in the HM patients at Pre-vac (n=28), TP1 (n=26), and TP2 (n=27). The threshold for antibody response was set as ≥4-fold increase in the geometric mean of the MFI value for the Pre-vac time point. The threshold values were set as follows, IgG S1: 5533, S2: 110710, RBD: 20946, Nucleocapside: 47556; IgA S1: 3105, S2: 9082, RBD: 12433, Nucleocapside: 10421; IgM S1: 4787, S2: 13983, RBD: 37083, Nucleocapside: 22803. Wilcoxon signed rank test adjusting p-values with the Bonferroni method, **** (p < 0.0001), *** (p < 0.001), ** (p < 0.01) and * (p ≤ 0.05). Numbers on top of each box plot represent the percentage of patients with a positive antibody response (above threshold for antibody response). **(B)** Plots showing the correlation between the levels of IgG antibody against SARS-CoV-2 Spike protein subunits S1, S2 and RDB and (left) SARS-CoV-2 Spike specific T cells responses and (right) sum of the estimated frequencies (%) for the SARS-CoV-2 Spike specific T cell responses for the HM patients at TP2. Only patients with positive IgG antibody levels were included. The Spearman correlation coefficient (r^2^) and p-values are indicated at the top of each plot. **(C)** Venn diagram showing a summary of the relationship between the SARS-CoV-2 Spike specific T cell responses and the IgG antibody responses against SARS-CoV-2 Spike S1 in the HM patients at TP2. **(D)** Box plots comparing the number of SARS-CoV-2 Spike-specific T cell responses (left) and the sum of their estimated frequencies (right) in HM patients at TP2 with (IgG+) and without (IgG-) IgG antibody response against SARS-CoV-2 Spike S1. Mann-Whitney test, IgG+ vs. IgG- for T cell responses (p = 0.399) and IgG+ vs. IgG- for sum of estimated frequencies (p=0.312).

**Figure 3:**
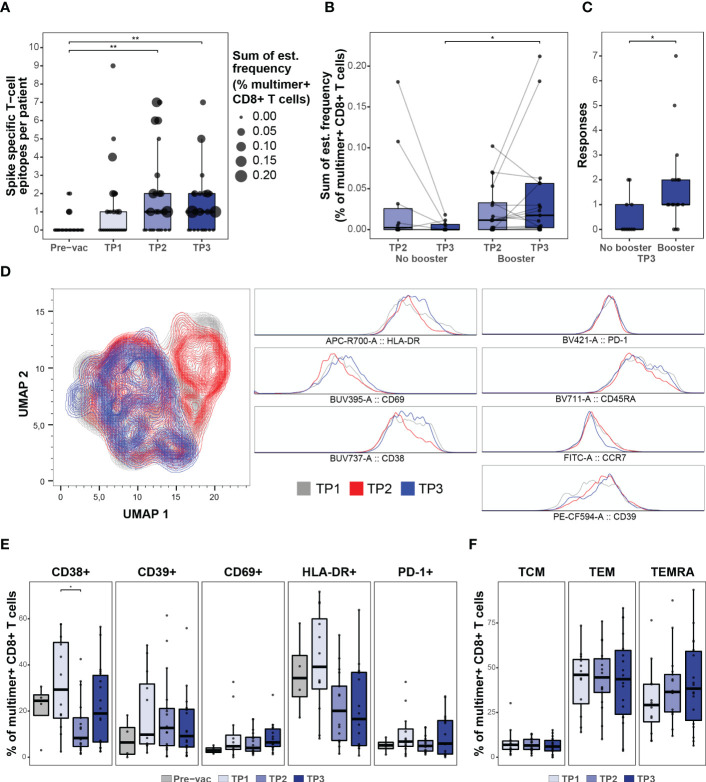
CD8 + T cell response after booster vaccination. **(A)** Summary of the number of SARS-CoV-2 Spike-specific T cell epitopes per individual in the HM patients before (Pre-vac, n=28) and after vaccination at TP1 (n=26), TP2 (n=27), and TP3 (n=27). The size of each dot is proportional to the sum of the estimated frequencies (%) for the significant responses in each individual. Wilcoxon signed rank test adjusting p-values with the Bonferroni method, Pre-vac vs. TP2 (p = 0.01) and Pre-vac vs. TP3 (p = 0.006). **(B)** Box plot comparing the estimated frequencies of the SARS-CoV-2 Spike-specific CD8+ T cells at TP2 and TP3 between HM patients vaccinated (booster; n=17) or not vaccinated (No booster; n=11) with a booster dose before TP3. Mann-Whitney test, TP3 no booster vs. TP3 booster (p = 0.02). **(C)** Box plot comparing the number of SARS-CoV-2 Spike-derived CD8+ T cell responses between booster and non-booster HM patients at TP3. Mann-Whitney test, no booster vs. booster (p = 0.04). **(D)** UMAP overlay (left) of SARS-CoV-2 Spike multimer^+^ T cells showing the clustering distribution at TP1, TP2, and TP3 according to the cell’s phenotype profile. Histogram overlay (right) comparing the expression of HLA-DR, CD69, CD38, PD-1, CD39, CD45RA and CCR7 cell surface markers in the SARS-CoV-2 Spike multimer^+^ T cells at TP1, TP2, and TP3 in the HM patients. **(E)** Box plots indicating the percentage of Spike pMHC multimer^+^ CD8^+^ T cells expressing the surface markers (CD38, CD39, CD69, HLA-DR and PD-1). Using the Mann-Whitney test adjusting p-values with the Bonferroni method, CD38^+^ TP1 vs TP2 (p=0.04). All other p-values were found to be non-significant. **(F)** Box plots showing the fraction of memory (TCM, TEM and TEMRA) Spike pMHC multimer^+^ CD8^+^ T populations based on the expression of CD45RA and CCR7 cell surface markers. Using the Mann-Whitney test adjusting p-values with the Bonferroni method, all p-values were found to be non-significant. * p ≤ 0.05, ** p ≤ 0.01.

**Figure 4:**
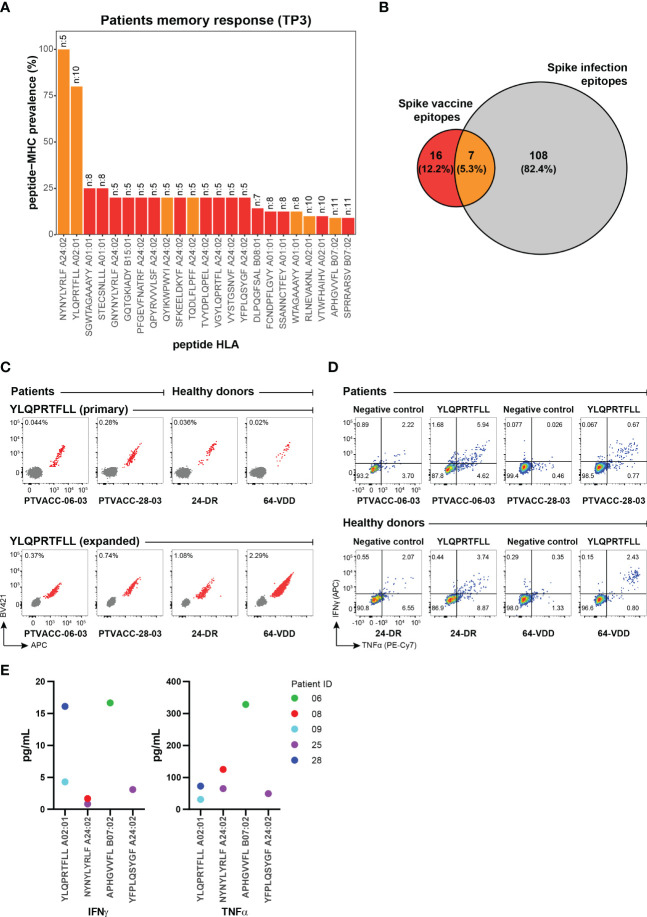
Vaccine-derived immunogenic epitopes establish functional and long-term T cell memory. **(A)** Prevalence of the vaccine-induced CD8^+^ T cell memory towards SARS-CoV-2 Spike epitopes detected in the HM patients at six months (TP3, n=27) post-vaccination. The number at the top of the bar represents the total number of patients analyzed for the corresponding pMHC specificity. Bars are colored according to the Venn diagram in panel **(B)**. **(B)** Venn diagram shows the number of CD8^+^ T cell epitopes unique to the BNT162b2 mRNA vaccine (red), and SARS-CoV-2 infection (grey) and those identified in both cases (orange). Numbers were obtained from the epitopes listed in **Supplementary Table S11**. **(C)** Combinatorial tetramer analysis of HLA-A02:01 restricted YLQPRTFLL immunodominant epitope in two HM patients (at TP3) and two healthy donors (at TP2) from PBMCs, directly stained *ex vivo* (top) and after *in vitro* expansion of PBMCs incubated with YLQPRTFLL peptides for two weeks (bottom). The number in the plot shows the percentage of CD8^+^ T cells binding to pMHC tetramers. **(D)** Functional evaluation of the Spike immunodominant epitope YLQPRTFLL-specific CD8^+^ T cells expanded from PBMCs of two HM patients (at TP3) and two healthy donors (at TP2) by intracellular cytokine staining after peptide stimulation and no stimulation (negative). Flow cytometry plots indicate the frequency (%) of CD8^+^ T cells double or single positive for TNF-α and INF-γ. **(E)** TNF-α and INF-γ concentrations (pg/mL) in cell culture supernatant from PBMCs from five HM patients after 24 hours stimulation with individual SARS-CoV-2 Spike epitopes. Four immunogenic epitopes identified at TP3 were selected for this analysis. Cytokine levels were measured by a Luminex bead-based multiplex assay.

T cell recognition data, determined by DNA-barcoded pMHC multimers analysis and Barracoda software, was plotted using RStudio version 4.1.0 (21). Peptide sequences with no significant enrichments are shown as gray dots and all peptides with a negative enrichment are set to LogFC equal zero (**Figure 1C**; **Supplementary Figure S2**). The ggplot2 package version 3.3.6 (22) was used to generate heatmap (**Supplementary Figure S6**), scatter (**Figure 2B**; **Supplementary Figure S4**) and box plots (**Figures 1E, F**, **2A, D**, **3A–C, 3E, F**; **Supplementary Figure S3**) for data visualization, and the venn diagrams shown in **Figures 2C**, **4B** were generated using the VennDiagram package version 1.7.3 (23). For statistical analysis, data was assumed to have a non-Gaussian distribution and non-parametric tests were therefore used. Wilcoxon signed rank test was used for single-paired comparisons and the Mann-Whitney test was used for unpaired comparisons. In cases where the Wilcoxon signed rank test was used to compare samples across different time points the p-values were adjusted using the Bonferroni method to correct for multiple comparisons. All statistical tests were performed using the rstatix package version 0.7.0 (24). The p-values are indicated in figure legends. The correlation coefficient (r2) and p-values shown in **Figure 2B** and **Supplementary Figure S4** were generated using the nonparametric, greater than zero Spearman’s correlation test as it was expected a positive correlation between the T-cell and the antibody response. Dot plots of the cytokine levels (**Figure 4E**) were generated using GraphPad Prism version 9.1.2 (GraphPad Software Inc., USA).

## Results

### Comprehensive longitudinal profiling of Spike-specific CD8^+^ T cells

We used DNA-barcoded peptide-major histocompatibility complex (pMHC) multimers to evaluate CD8^+^ T cell response to the BNT162b2 mRNA vaccine, including the impact of booster immunization, in patients with pre-existing hematological cancers (CLL, n=23; MDS, n=5; **Supplementary Table S1**) up to six months post-vaccination and compared with a cohort of healthy individuals (n=19; **Supplementary Table S2**) (**Figure 1A**). By using NetMHCpan4.1 (25), we selected 415 unique peptides (8-11 amino acids) spanning the complete SARS-CoV-2 Spike protein encoded by the BNT162b2 mRNA vaccine (GenBank ID: QHD43416.1). The peptides were predicted to bind one or more of the nine prevalent HLA-A and HLA-B molecules generating 506 peptide-HLA pairs for experimental evaluation (**Figure 1B**; **Supplementary Table S5**). Additionally, 67 peptides from cytomegalovirus (CMV), Epstein-Barr viral (EBV), and influenza (FLU), (together denoted as CEF) were included to compare Spike to CEF-antigen-reactive T cells (**Supplementary Table S6**). DNA-barcoded multimers were prepared by loading HLA-specific peptides on MHC molecules and multimerized on dextramer backbone tagged with DNA-barcodes, providing each pMHC multimer with a unique DNA-barcode (17). PBMCs were incubated with HLA-matching (**Supplementary Tables S3** and **S4**) pMHC multimers and stained with a phenotype antibody panel to identify multimer-reactive CD8^+^ T cells (**Supplementary Figure S1**) (26).

### BNT162b2 mRNA vaccine induces persistent antigen-specific CD8^+^ T cells in CLL and MDS patients

BNT162b2 mRNA vaccine-induced Spike antigen-specific CD8^+^ T cells were detected in a substantial fraction of CLL and MDS patients already between days 7-10 after the first dose of vaccination (TP1). The frequency and total number of Spike-specific T cell responses further increased after the second vaccination (TP2) and persisted up to six months (TP3) following the initial vaccination (**Figure 1C**; **Supplementary Figure S2A** (top); **Supplementary Table S8**). Altogether, we identified 59 immunogenic responses of the 506 tested pMHC specificities across all time points (**Supplementary Table S9**). Most of the T cell responses were identified as HLA-A01:01 (10 responses), HLA-A02:01 (11 responses), and HLA-A24:02 (22 responses) restricted Spike peptides (**Figure 1D**). CEF epitope-specific CD8^+^ T cells were identified in samples from all four time points with no observed changes pre- to post-SARS-CoV-2 vaccination, except for a rise in influenza epitope-specific CD8^+^ T cells in a few patients, likely resulting from influenza vaccination (**Figure 1C**; **Supplementary Figure S2A** (bottom); **Supplementary Table S1**).

Strikingly, a vaccine-driven enrichment of CD8^+^ T cells was detected in the patient group with a significantly higher frequency of Spike-specific CD8^+^ T cells compared to a healthy donor cohort evaluated at a similar time point after the second vaccination (**Supplementary Figure S2B**; **Supplementary Table S10**). Also, the number of Spike-derived epitopes recognized in the patient group appeared higher, although not significant (**Figure 1E**). Overall, after two doses of vaccination (at TP2), Spike-specific CD8^+^ T cells were detected in 17 out of 28 patients and 10 out of 19 healthy donors (**Supplementary Figures S2A, B**). No difference was observed in the vaccine-specific T cell responses between CLL and MDS patients (**Supplementary Figure S3**). Phenotypic characterization, based on the cell surface markers (CD38, CD39, CD69, HLA-DR, and PD-1) of multimer^+^ SARS-CoV-2 Spike-specific CD8^+^ T cells, displayed a signature of T cell activation in both cohorts, while a significantly higher fraction of such cells expressed CD38 and PD-1 in the healthy donors (**Figure 1F**). We next compared the T cell immunity with the vaccine-driven humoral immunity by analyzing plasma levels of IgG, IgM, and IgA antibodies. In contrast to the early detection of vaccine-induced T cells (**Figure 1C**), the antibody levels remained low at TP1, and significantly increased only after the second vaccination at TP2 (**Figure 2A**). Compared to TP1, at TP2 level of IgG antibodies increased the most including the fraction of individuals with a positive antibody response (Spike S1 and S2 20% to 73.1%, and RBD 20% to 69.2%) followed by IgA (Spike S1 16% to 50%, S2 20% to 69.2%, and RBD 24% to 53.8%) and IgM (Spike S1 24% to 50%, S2 16% to 34.6%, and RBD 20% to 42.3%) antibodies respectively. Since antibody levels were highest at TP2 and IgG levels increased the most, we next compared the association between the level of vaccine-specific T-cells and IgG antibodies. We found a significant association between the antibody levels (IgG) and the level of vaccine-specific CD8^+^ T-cells (number of responses and their estimated frequencies) in all the patients that had an antibody response (n=19, **Figure 2B**). However, no association was observed when compared for all the patients analyzed at TP2 (n=27, **Supplementary Figure S4A**), or patients with both an antibody and a T-cell response (n=11, **Supplementary Figure S4B**), or all patients with a T-cell response (n=17, **Supplementary Figure S4C**). Furthermore, at TP2 41% of the cohort had both T cell and Spike S1-specific IgG antibody responses, compared to 22% and 30% of individuals with only T cell response or with only antibody response, respectively (**Figure 2C**). Additionally, we didn’t find any increase in the vaccine-specific T-cells in patients without IgG antibodies compared to patients with IgG antibodies at TP2 (**Figure 2D**).

### Booster vaccination maintains vaccine-induced long-term CD8^+^ T cell memory

Once established, the vaccine-induced SARS-CoV-2 antigen-specific T cell immunity remained long-term (at TP3, six months post-vaccination) with a median of one T cell response and the frequency of multimer^+^ T cells reaching up to 0.2% of the total CD8^+^ T cells in the blood (**Figure 3A**). Importantly, a booster vaccination was required to maintain a higher frequency of vaccine-induced CD8^+^ T cells. Patients without a booster vaccination (n=11) showed an overall decline in the Spike antigen-specific CD8^+^ T cell frequency from TP2 to TP3, whereas a significantly higher frequency, and breadth (total number of Spike-epitope recognized), was observed in patients vaccinated with a booster dose (n=17) prior to TP3 (**Figures 3B, C**), suggesting a positive impact of the booster vaccine in this patient group. Post-vaccination longitudinal analysis of antigen-specific CD8^+^ T cells phenotype in HM patients further revealed an early activation of vaccine-induced T cells (**Figures 3D, E**; **Supplementary Figure S5**) followed by a transition to circulating memory T cells (CD45RA^+^ CCR7^-^) (**Figures 3D, F**; **Supplementary Figure S5**), which could be essential for T cell-mediated long-term protection (27, 28).

At six-months post-vaccination, the long-term memory was established by a total of 23 Spike-derived epitopes and a strong immunodominance was observed for NYNYLYRLF (100% prevalence) and YLQPRTFLL (80% prevalence) epitopes restricted to HLA-A24:02 and HLA-A02:01 respectively (**Figure 4A**; **Supplementary Figure S6**). These two epitopes were among the 7 immunogenic peptides that showed T cell activation after vaccination and natural infection (26, 29–32), thus, likely to provide immune protection in SARS-CoV-2 infection (**Figure 4B**) (33, 34). Furthermore, even with an overall narrow CD8^+^ T cell repertoire compared to natural infection, 16 of the Spike-derived immunogenic epitopes were vaccine-unique across the nine HLAs tested in this study (**Figure 4B**; **Supplementary Table S11**) and requires further evaluation of their impact on disease protection.

To evaluate the effectiveness of antigen-specific memory T cells, we validated *ex vivo* frequencies of CD8^+^ T cells reactive to several Spike and CEF epitopes using fluorophore labeled pMHC tetramers (**Supplementary Figure S7**) and showed comparable antigen-specific expansion of vaccine-induced CD8^+^ T cells in HM patients and healthy donors (**Figure 4C**). The expanded YLQPRTFLL-HLA-A02:01-reactive CD8^+^ T cells were functionally active upon peptide stimulation and the cytokine (IFN-γ and TNF-α) secretion profiles were comparable between HM patients and healthy donors (**Figure 4D**; **Supplementary Figure S8**). We next quantified the levels of IFN-γ and TNF-α release upon peptide stimulation to functionally characterize vaccine-specific CD8^+^ T-cells of different antigen specificities in HM patients. Patient-derived CD8^+^ T-cells (from five patients at TP3) restricted to the two immunodominant epitopes (YLQPRTFLL and NYNYLYRLF) as well as to two other immunogenic epitopes showed varying levels of IFN-γ and TNF-α secretion upon stimulation with respective peptides (**Figure 4E**). These results provide important indication on the functionality of vaccine-induced CD8^+^ T-cells in HM patients. Altogether, our data shows the presence of functionally active long-lasting memory CD8^+^ T cells in HM patients vaccinated with the BNT162b2 mRNA vaccine.

## Discussion

An ongoing concern during the COVID-19 pandemic has been the severe disease outcome experienced by patients with comorbidities, especially patients with hematological malignancies, which have shown increased COVID-19 disease-related complications and mortality (4–6). Such patients have been prioritized for primary prevention by COVID-19 vaccination; however, few data are available on their efficacy in this vulnerable population. Here we present a detailed characterization of the CD8^+^ T cell immunogenicity, its long-term persistence, and its correlation with antibody response in patients with CCL and MDS who received up to three doses of the BNT162b2 mRNA vaccine.

Our longitudinal analysis revealed an early activation of vaccine-specific CD8^+^ T cell response just 7-10 days after the first vaccine dose. The number and frequency of responses further increased after the second vaccine dose and persisted up to six months. The frequency of vaccine-induced antigen-specific CD8^+^ T cells in the patient cohort was unexpectedly higher compared to the healthy donor group after the second vaccine dose. These data correlate with an increased T cell activation observed after SARS-CoV-2 infection in hematological cancer patients, especially in severe COVID-19 disease (26, 27). Furthermore, vaccine-induced CD8^+^ T cell results in our healthy donors are in-line with the existing studies. *Naranbhai et al.* showed <50% of healthy individuals showing a CD8^+^ T cell response across three different vaccines (35). Similarly, existing data shows a heterogenous CD8^+^ T cell response in healthy SARS-CoV-2 naïve individuals after two doses of mRNA vaccine, and vaccine-specific CD8^+^ T cells were detected at low frequency with progressive increase over time (35–38).

Since an age-dependent reduction in SARS-CoV-2 vaccine-specific T cell activation has been shown previously (39), a higher T cell response in our patient group (median age 71.8 years) compared to a younger healthy donor group (median age 42.2 years) further signifies these data. However, it’s important to emphasize that our healthy cohort samples were collected from a different demographic location (Italy) than the patient cohort (Denmark). Although, prior studies have shown comparable vaccine-induced humoral responses for healthy individuals and hematological patients from these two locations (40, 41). Overall, the majority of studies have shown substantially lower humoral response in hematological cancer patients compared to healthy individuals. Other confounding factors between these two groups may influence the vaccine-specific immune response that we have not evaluated in this study due to limited data availability and the cohort size (42).

Patients with hematological malignancies have shown limited induction of humoral response after the first dose of the BNT162b2 mRNA vaccine, and the antibody response rates range from 27 to 87.3% after the second dose depending on the type of disease and anticancer therapy received (35, 39, 41–44). Similar to these previous results, in our study the antibody response (IgG) in the CLL and MDS patients had a significant increase only after the second vaccine dose with a response rate of 73.1%. Interestingly, after two doses of vaccination patients with an antibody response strongly correlated for a vaccine-specific T cell response. However, we didn’t observe a compensatory correlation for T-cell activation in patients with no antibody response, although some patients could elicit a T cell response. Significance of these T cell responses in protection and disease outcome in patients without an antibody response need further evaluation.

Studies on BNT162b2 vaccine-induced T-cell responses in patients with hematological malignancies have reported heterogeneous response rates from 27% to 86% measured between 14 days to 6 weeks after full vaccination with 2 doses (43–48). Responses varied according to the type of malignancy and cancer treatment received. Patients after allogeneic hematopoietic cell transplantation reported the lowest vaccine T cell response rates (27%) (43). In the case of CLL patients, a study showed that 53% of the individuals elicited a T-cell response against the Spike protein after the second vaccine dose (day 42 from the first dose) (44). Our patient cohort consists of CLL and MDS patients that didn’t receive any immunomodulatory treatment and T cell responses was detected in 63% of the patients after two vaccine doses.

The analysis of the impact of booster immunization in this study showed that a third-dose BNT162b2 vaccine is required to maintain the long-term vaccine-induced CD8^+^ T cell memory responses in this immunocompromised patient population. Similar results were reported by Lim et al. (49) in patients with lymphoma, vaccinated either with ChAdOx1 nCoV-19 (ChAdOx1) or BNT162b2 vaccine, where 63% of patients displayed antigen-specific T-cell responses, which further increased after a third dose irrespective of their cancer treatment.

These previous studies have analyzed T –cell immunity to COVID-19 mRNA vaccination in patients with hematological malignancies by intracellular cytokine staining and flow cytometry, or by measurement of secreted IFN-γ/IL-2 by ELISpot or FluoroSpot techniques. Through our T cell screening strategy using DNA-barcoded pMHC multimers, we were able to map the CD8^+^ T cell recognition and identified 59 BNT162b2 vaccine-derived immunogenic epitopes. Of these, 23 epitopes established long-term CD8^+^ T cell memory response maintained up to six-months post-vaccination. Importantly, 16 of these epitopes were vaccine-unique and have not been identified in inducing CD8^+^ T cells in natural infection based on the published literature. Immunodominance was observed for two of the epitopes: HLA-A24:02- NYNYLYRLF and HLA-A02:01-YLQPRTFLL, which have also been reported after natural infection. T cell immunogenicity mapping can provide valuable information for vaccine design for this patient group.

## Data availability statement

The original contributions presented in the study are included in the article/**Supplementary Material**. Further inquiries can be directed to the corresponding author.

## Ethics statement

The studies involving human participants were reviewed and approved by the Committee on Health Research Ethics in the Capital Region of Denmark and the ethics committee at National Cancer Institute Foundation Pascale, Napoli, Italy. The patients/participants provided their written informed consent to participate in this study.

## Author contributions

SA designed and performed the experiments, analyzed the data, and wrote the manuscript. DS, designed the research, facilitated, and collected patient samples, analyzed clinical data, and wrote the manuscript. KM analyzed the data, prepared the figures, and wrote the manuscript. TT and DT designed and performed experiments. MT coordinated and facilitated clinical samples. AG and LB supervised the clinical study, patient participation, clinical data, and sample collection, and wrote the manuscript. SH conceived the idea, designed, and supervised the study, and wrote the manuscript. SS conceived the idea, designed and supervised the study, designed and performed the experiments, analyzed the data, and wrote the manuscript. All authors contributed to the article and approved the submitted version.
