# Supplementary material for: Three doses of BNT162b2 COVID-19 mRNA vaccine establish long-lasting CD8+ T cell immunity in CLL and MDS patients
Source: Front Immunol. 2023 Jan 10;13:1035344. doi: 10.3389/fimmu.2022.1035344 (PMC9873231; doi:10.3389/fimmu.2022.1035344)
Supplement: Supplementary file 1 [file DataSheet_1.docx]

**Three-doses of BNT162b2 COVID-19 mRNA vaccine establishes long-lasting CD8**^+^ **T cell immunity in CLL and MDS patients**

Susana Patricia Amaya Hernandez^1,4^, Ditte Stampe Hersby^2,4^, Kamilla Kjærgaard Munk^1^, Tripti Tamhane^1^, Darya Trubach^1^, Maria Tagliamonte^3^, Luigi Buonaguro ^3^, Anne Ortved Gang^2^, Sine Reker Hadrup^1^, Sunil Kumar Saini^1,*^

**Affiliation of Institutions**

^1^Department of Health Technology, Section of Experimental and Translational Immunology, Technical University of Denmark, Kongens Lyngby, Denmark

^2^Department of Hematology, Copenhagen University Hospital, Rigshospitalet, Copenhagen, Denmark

^3^Innovative Immunological Models Unit, National Cancer Institute Pascale Foundation – IRCCS, Napoli, Italy

^4^These authors contributed equally

^*^Correspondance: [sukusa@dtu.dk](mailto:sukusa@dtu.dk) (S.K.S.)

**Supplementary Figures**

| 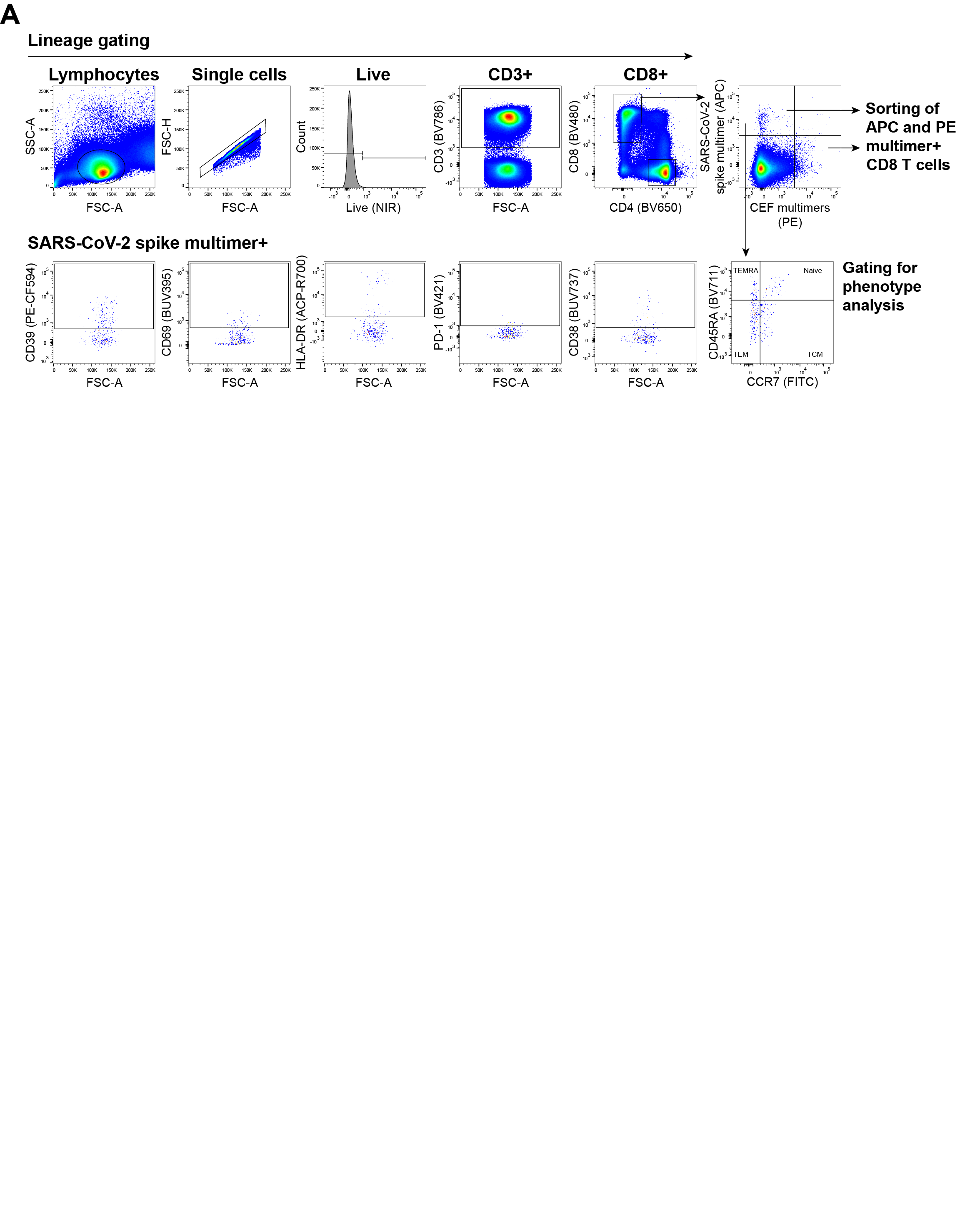 |
| --- |
| **Supplementary Figure S1. Gating strategy for sorting pMHC multimer^+^ CD8^+^ T cells and phenotype analysis.** Representative flow cytometry plots for gating strategy on HM patients and healthy donors PBMCs stained with DNA-barcoded pMHC multimers and surface antibody markers to sort SARS-CoV-2 Spike (APC) and CEF (PE) multimer^+^ CD8^+^ T cells and to quantify multimer^+^ CD8^+^ T cells expressing phenotype markers. |
| 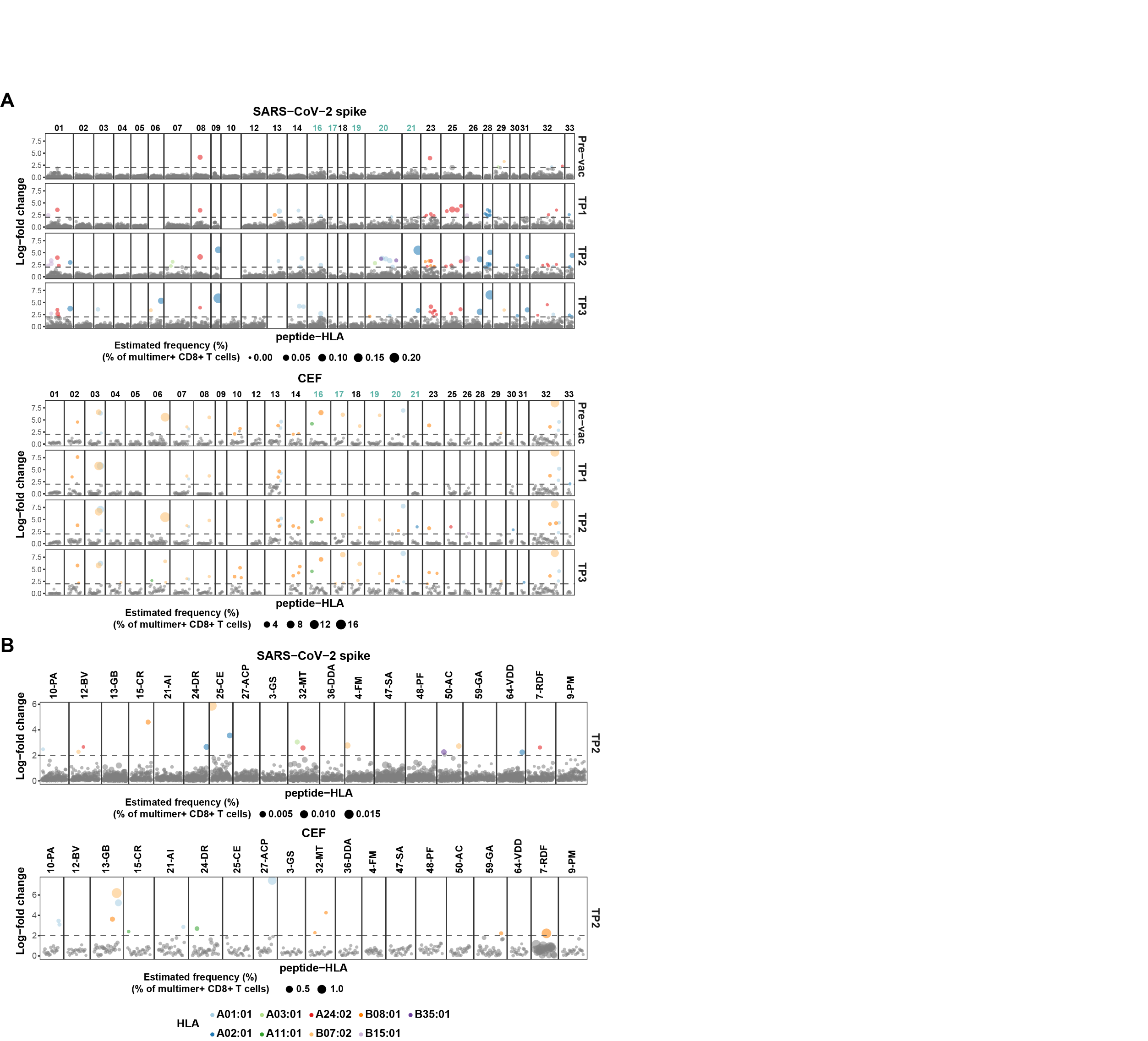 |
| **Supplementary Figure S2. Summary of the CD8^+^ T cell recognition in HM patients and healthy donors.** (**A**) CD8^+^ T cell recognition to SARS-CoV-2 Spike- (**top**) and CEF-derived peptides (**bottom**) in each of the HM patients before (Pre-vac (n=28)) and after vaccination (TP1 (n=26), TP2 (n=27) and TP3 (n=27)). Patient IDs in green color represent MDS patients, rest are CLL patients. (**B**) CD8^+^ T cell recognition to SARS-CoV-2 Spike- (**top**) and CEF-derived peptides (**bottom**) in each of the healthy donors after vaccination at TP2 (n=19). Each dot represents one peptide-HLA combination per sample, their size is proportional to the estimated frequency (%) calculated from the percentage read count of the associated barcode out of the percentage of CD8^+^ multimer^+^ T cells, and they are colored according to their HLA. |

| 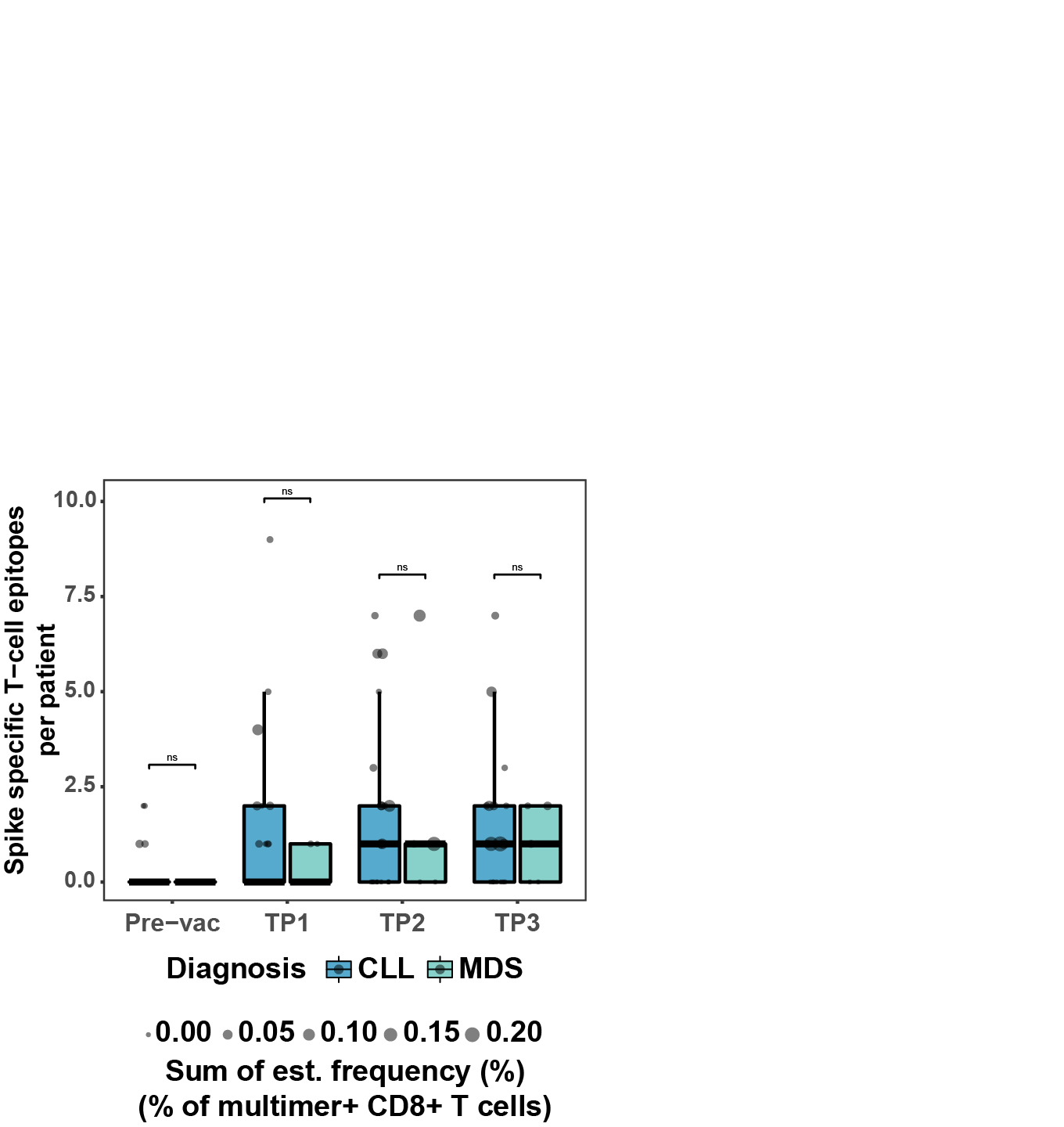 |
| --- |
| **Supplementary Figure S3. Multimer^+^ CD8^+^ T cell analysis.** Box plot compares the number of SARS-CoV-2 Spike specific T cell epitopes per patient between CLL and MDS diagnosis across the four time points. The size of the dots is proportional to the sum of the estimated frequencies (%) of multimer^+^ CD8^+^ T cells for the significant responses in each individual. Mann-Whitney test, Pre-vac CLL vs. MDS (p = 0.349), TP1 CLL vs. MDS (p = 0.498), TP2 CLL vs. MDS (p = 0.796), TP3 CLL vs. MDS (p = 0.870). |

| 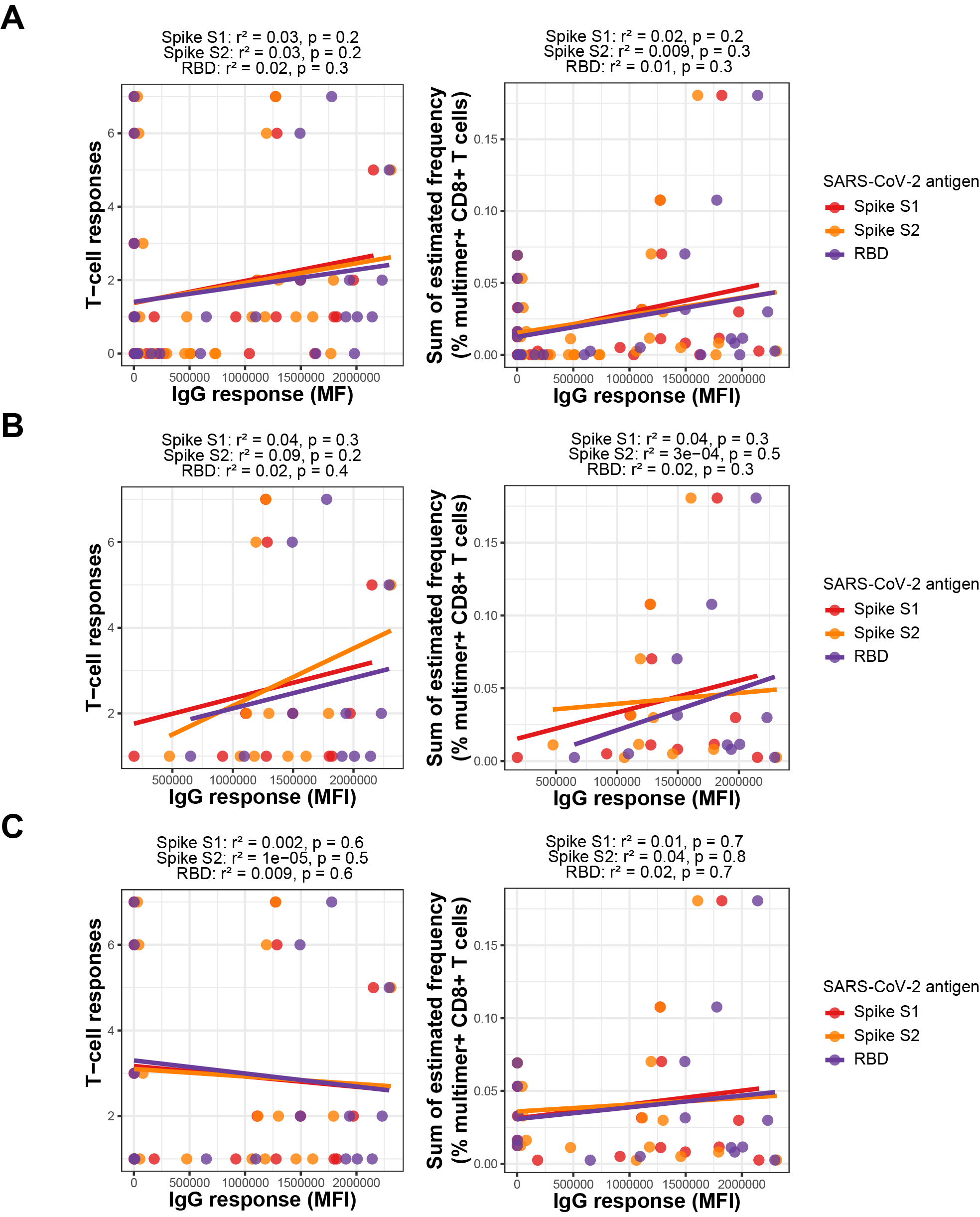 |
| --- |
| **Supplementary Figure S4. Correlation between IgG antibody levels and T cells responses.** Plots showing the correlation between SARS-CoV-2 Spike specific T cells (**Left**, the total number of responses; **Right**, estimated frequencies) and the levels of IgG antibody against SARS-CoV-2 Spike protein subunits (S1, S2, and RDB ). (**A**) all HM patients analyzed at TP2 (**B**) HM patients positive for both IgG antibody and T cell response at TP2, and (**C**) HM patients with positive T cell response at TP2. The Spearman correlation coefficient (r^2^) and p-values are indicated at the top of each plot. |

| **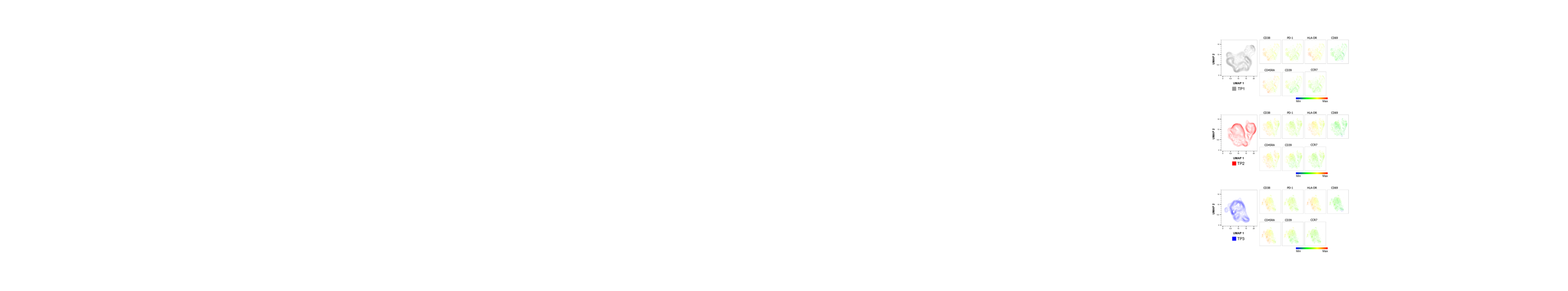** |
| --- |
| **Supplementary Figure S5. UMAP visualization of SARS-CoV-2 Spike specific CD8+ T cells phenotype.** UMAP plots showing the expression of individual markers CD38, PD-1, HLA-DR, CD69, CD45-RA, CD39 and CCR7 for SARS-CoV-2 pMHC multimers+ CD8^+^ T cells in HM patients at TP1, TP2 and TP3. |

| 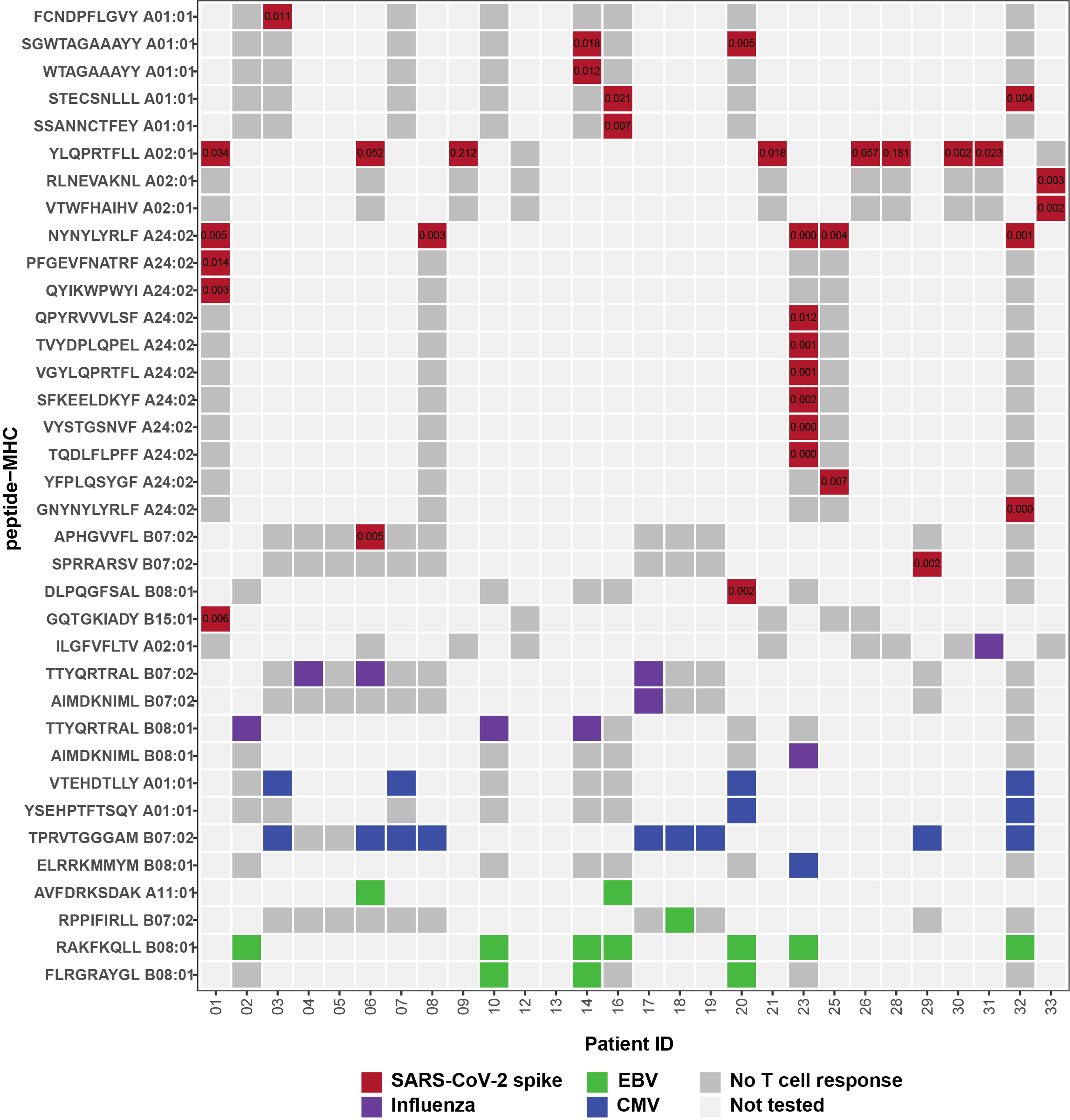 |
| --- |
| **Supplementary Figure S6 Heatmap plot with patient-specific long-term memory CD8^+^ T cell responses.** Summary of SARS-CoV-2 Spike and CEF-specific epitopes identified in HM patients at TP3. Numbers on the plots show the estimated frequencies of SARS-CoV-2 antigen-specific CD8^+^ T-cells identified in individual patients. Sample PTVACC-13-04 was not included in the analysis. |

| 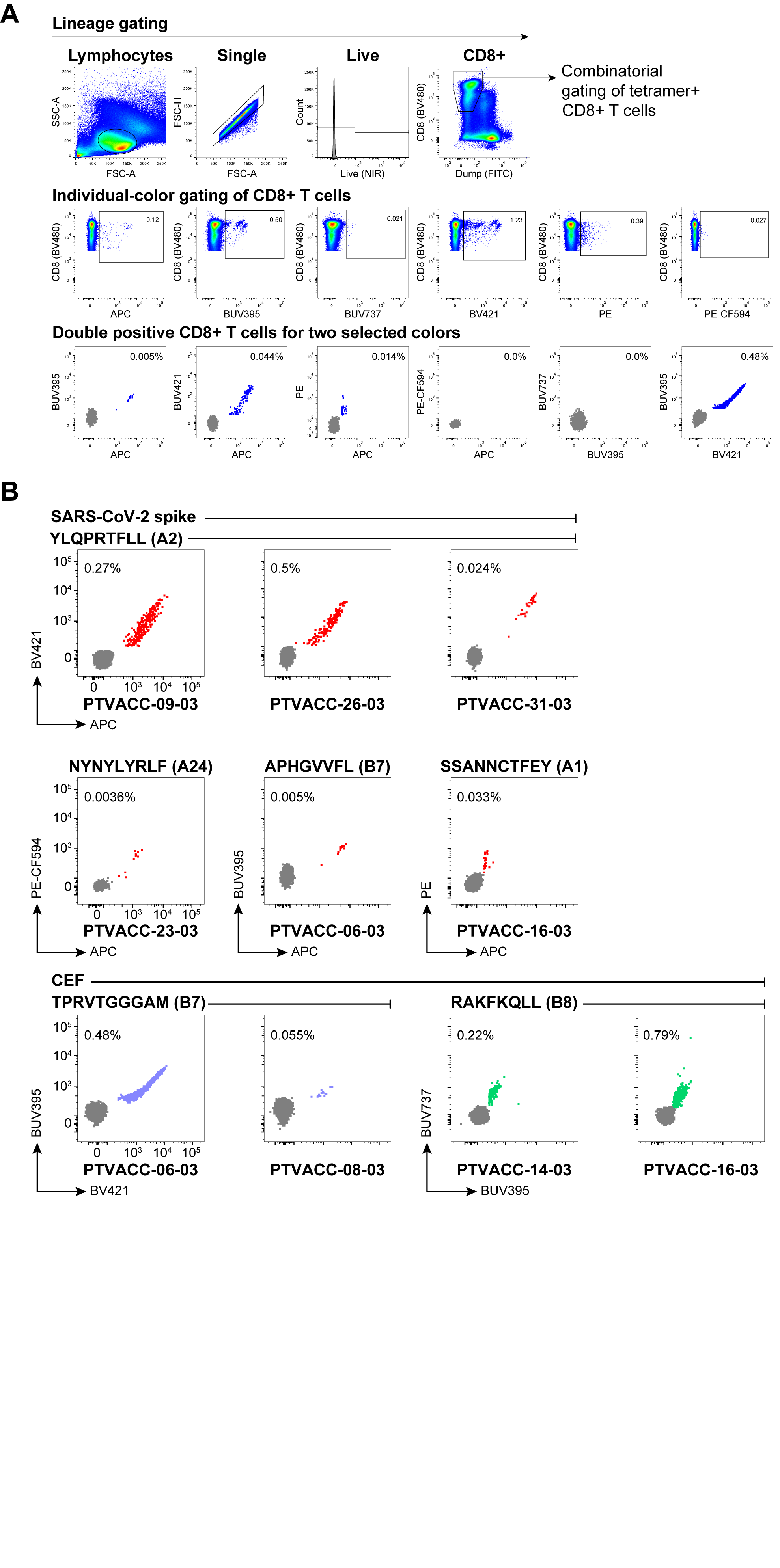 |  |
| --- | --- |
| **Supplementary Figure S7. Validation of selected SARS-CoV-2 Spike and CEF epitopes using pMHC tetramers.** (**A**) Representative flow cytometry plots with gating strategy to identify SARS-CoV-2 Spike- and CEF- specific T cells using combinatorial fluorescently labeled pMHC tetramers. Individual-color gating of CD8^+^ T cells was used to select double-positive cells in two tetramer colors and negative in the remaining colors. (**B**) Combinatorial tetramer analysis in HM patients (TP3) of SARS-CoV-2 Spike and CEF derived epitopes identified by DNA barcoded multimers analysis. |  |
| 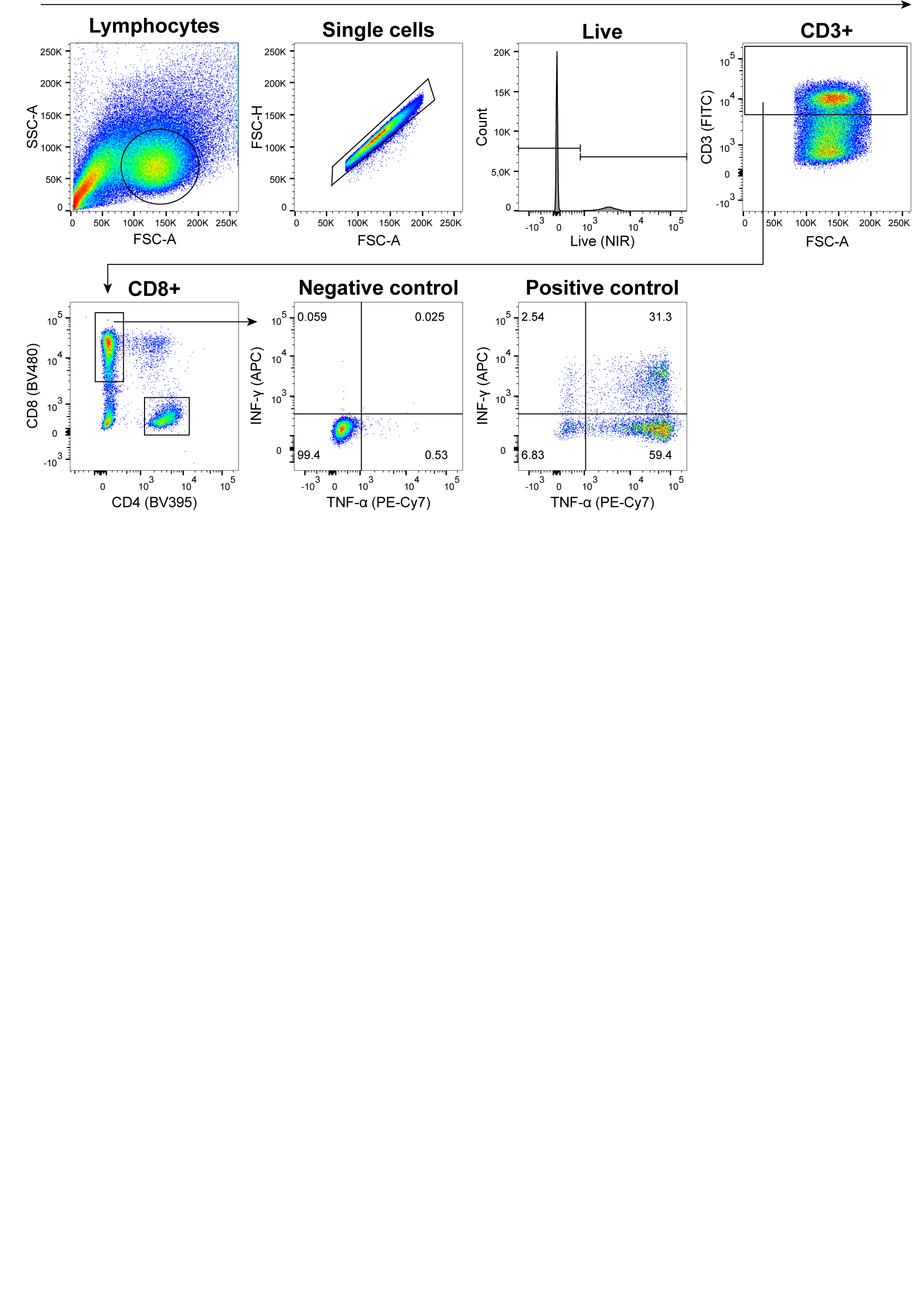 | |
| **Supplementary Figure S8. Gating strategy for the functional evaluation of SARS-CoV-2 Spike specific CD8^+^ T cell responses in HM patients.** Representative flow cytometry plots showing the gating strategy to measure intracellular cytokines IFN-γ, and TNF-α of expanded T cells upon stimulation with YLQPRTFLL (HLA-A0201) peptide. Cells incubated with no peptide were used as negative control and cells incubated with a leukocyte activation cocktail were used as a positive control. The numbers on the plot indicate the frequency (%) of CD8^+^ T cells positive for the analyzed cytokines. | |

**Supplementary Tables**

**Supplementary Table S1. Patient cohort information**

| **S.No.** | **Patient ID** | **Age** | **Gender** | **Diagnosis** | **Current treatment** | **Previous treatment** | **Comorbidity** | **BNT162b2 COVID-19 vaccine** | | | **Influenza vaccine** | | **Blood sample collection** | | | |
| --- | --- | --- | --- | --- | --- | --- | --- | --- | --- | --- | --- | --- | --- | --- | --- | --- |
|  |  |  |  |  |  |  |  | **Dose 1** | **Dose 2** | **Booster** | **Date** | **Type** | **Pre-vac** | **TP1** | **TP2** | **TP3** |
| 1 | PTVACC-01 | 65 | M | CLL |  |  | Previous prostate cancer, AIHA. | 26-04-21 | 25-05-21 | 11-09-21 |  |  | 04-03-21 | 21-05-21 | 25-06-21 | 04-11-21 |
| 2 | PTVACC-02 | 70 | M | CLL |  |  |  | 12-04-21 | 08-05-21 |  | 01-10-20 | Influvactetra | 04-03-21 | 22-04-21 | 11-06-21 | 12-10-21 |
| 3 | PTVACC-03 | 66 | F | CLL |  |  |  | 13-03-21 | 09-04-21 |  | 14-10-20 | Vaxigriptetra | 04-03-21 | 22-03-21 | 28-05-21 | 14-09-21 |
| 4 | PTVACC-04 | 76 | M | CLL |  |  |  | 13-04-21 | 04-05-21 | 11-09-21 | 08-10-20 | Influvactetra | 04-03-21 | 26-04-21 | 11-06-21 | 12-10-21 |
| 5 | PTVACC-05 | 75 | M | CLL | Substitution of gammaglobulin every 10th day during the winter | Rituximab (2013) due to AIHA |  | 06-03-21 | 01-04-21 |  | 05-10-20 | Influvactetra | 04-03-21 | 15-03-21 | 10-05-21 | 14-09-21 |
| 6 | PTVACC-06 | 79 | M | CLL |  | Rectal cancer (2012), treated with RT, CT and surgery. Apoplexy (2020) |  | 19-03-21 | 09-04-21 | 15-09-21 | 26-10-20 | Vaxigriptetra | 04-03-21 |  | 07-05-21 | 06-10-21 |
| 7 | PTVACC-07 | 78 | F | CLL |  |  |  | 19-03-21 | 09-04-21 |  | 08-10-20 | Influvactetra | 04-03-21 | 26-03-21 | 21-05-21 | 14-09-21 |
| 8 | PTVACC-08 | 73 | F | CLL | Substitution of gammaglobulin every 10th day during the winter |  |  | 11-04-21 | 05-05-21 | 11-09-21 | 04-11-20 | Influvactetra | 04-03-21 | 19-04-21 | 11-06-21 | 12-10-21 |
| 9 | PTVACC-09 | 76 | F | CLL |  |  |  | 13-04-21 | 08-05-21 | 15-09-21 | 07-10-20 | Influvactetra | 04-03-21 | 22-04-21 | 18-06-21 | 12-10-21 |
| 10 | PTVACC-10 | 83 | M | CLL |  |  |  | 21-03-21 | 14-04-21 |  | 07-10-20 | Vaxigriptetra | 04-03-21 |  |  | 24-09-21 |
| 11 | PTVACC-12 | 68 | F | CLL |  |  |  | 05-03-21 | 31-03-21 |  |  |  | 04-03-21 | 15-03-21 | 07-05-21 | 14-09-21 |
| 12 | PTVACC-13 | 83 | F | CLL |  |  | Diabetes, previous breast cancer. | 07-03-21 | 02-04-21 |  |  |  | 04-03-21 | 15-03-21 | 10-05-21 | 14-09-21 |
| 13 | PTVACC-14 | 62 | M | CLL |  |  |  | 12-05-21 | 23-06-21 | 03-11-21 | 05-10-21 | Vaxigriptetra | 10-03-21 | 19-05-21 | 13-07-21 | 16-11-21 |
| 14 | PTVACC-16 | 73 | M | MDS (CMML) | Deferasirox. Regular blood transfusions. |  |  | 10-04-21 | 05-05-21 | 13-09-21 | 09-10-20 | Vaxigriptetra | 10-03-21 | 19-04-21 | 18-06-21 | 07-10-21 |
| 15 | PTVACC-17 | 74 | F | MDS | Deferasirox. Regular blood transfusions. |  |  | 18-04-21 | 13-05-21 | 11-09-21 | 05-10-20 | Influvactetra | 10-03-21 | 29-04-21 | 18-06-21 | 26-10-21 |
| 16 | PTVACC-18 | 83 | M | CLL |  |  |  | 18-03-21 | 12-04-21 |  | 09-10-20 | Vaxigriptetra | 10-03-21 | 26-03-21 | 04-06-21 | 24-09-21 |
| 17 | PTVACC-19 | 69 | F | MDS | Deferasirox. Regular blood transfusions. |  | Azacitidine, erythropoietin. | 10-04-21 | 01-05-21 | 13-09-21 |  |  | 10-03-21 | 19-04-21 | 18-06-21 | 07-10-21 |
| 18 | PTVACC-20 | 73 | F | MDS |  | Interferon |  | 18-04-21 | 13-05-21 |  | 09-10-20 | Vaxigriptetra | 10-03-21 | 26-04-21 | 25-06-21 | 16-11-21 |
| 19 | PTVACC-21 | 78 | F | MDS | Deferasirox. Regular blood transfusions. | Revlimid |  | 08-04-21 | 06-05-21 |  | 02-10-20 | Influvactetra | 10-03-21 | 21-04-21 | 16-06-21 | 06-10-21 |
| 20 | PTVACC-23 | 78 | M | CLL |  | Rituximab + Bendamustin (last treatment sep 2020) | Diabetes | 29-03-21 | 20-04-21 | 12-09-21 |  |  | 17-03-21 | 07-04-21 | 28-05-21 | 24-09-21 |
| 21 | PTVACC-25 | 70 | F | CLL |  |  |  | 18-04-21 | 09-05-21 | 10-09-21 | 07-10-20 | Vaxigriptetra | 22-03-21 | 26-04-21 | 11-06-21 | 07-10-21 |
| 22 | PTVACC-26 | 54 | M | CLL |  |  |  | 25-05-21 | 01-07-21 | 17-09-21 |  |  | 09-04-21 | 04-06-21 | 12-08-21 | 16-11-21 |
| 23 | PTVACC-28 | 67 | M | CLL |  |  |  | 27-04-21 | 01-06-21 | 22-09-21 | 16-10-20 | Vaxigriptetra | 16-04-21 | 04-05-21 | 25-06-21 | 02-11-21 |
| 24 | PTVACC-29 | 59 | M | CLL |  |  |  | 11-05-21 | 16-06-21 | 03-11-21 |  |  | 23-04-21 | 19-05-21 | 13-07-21 | 16-11-21 |
| 25 | PTVACC-30 | 74 | M | CLL |  |  |  | 10-04-21 | 04-05-21 | 14-09-21 | 12-10-20 | Influvactetra | 09-04-21 | 19-04-21 | 08-06-21 | 07-10-21 |
| 26 | PTVACC-31 | 62 | F | CLL |  |  |  | 09-05-21 | 16-06-21 | 23-09-21 |  |  | 16-04-21 | 19-05-21 | 09-07-21 | 16-11-21 |
| 27 | PTVACC-32 | 72 | F | CLL |  | Rituximab + Bendamustin (2019) |  | 17-04-21 | 09-05-21 | 14-09-21 | 02-10-20 | Vaxigriptetra | 14-04-21 | 26-04-21 | 21-06-21 | 07-10-21 |
| 28 | PTVACC-33 | 71 | F | CLL |  |  |  | 17-04-21 | 08-05-21 |  | 07-10-20 | Influvactetra | 16-04-21 | 26-04-21 | 18-06-21 | 12-10-21 |

**Supplementary Table S2. Healthy donors information**

| **S. No.** | **Healthy donor ID** | **Age** | **Gender** | **BNT162b2 COVID-19 vaccine** | | **Blood sample collection** |
| --- | --- | --- | --- | --- | --- | --- |
|  |  |  |  | **Dose 1** | **Dose 2** | **TP2** |
| 1 | 3-GS | 34 | M | 27-12-20 | 17-01-21 | 28-01-21 |
| 2 | 4-FM | 35 | M | 27-12-20 | 17-01-21 | 02-02-21 |
| 3 | 7-RDF | 50 | F | 31-12-20 | 21-01-21 | 05-02-21 |
| 4 | 9-PM | 66 | M | 31-12-20 |  | 20-01-21 |
| 5 | 10-PA | 35 | F | 01-01-21 | 22-01-21 | 08-02-21 |
| 6 | 12-BV | 40 | F | 02-01-21 | 23-01-21 | 10-02-21 |
| 7 | 13-GB | 35 | F | 05-01-21 | 26-01-21 | 12-02-21 |
| 8 | 15-CR | 65 | F | 08-01-21 | 29-01-21 | 10-02-21 |
| 9 | 21-AI | 50 | M | 08-01-21 | 28-01-21 | 08-02-21 |
| 10 | 24-DR | 38 | F | 08-01-21 | 29-01-21 | 15-02-21 |
| 11 | 25-CE | 64 | F | 08-01-21 | 29-01-21 | 12-02-21 |
| 12 | 27-ACP | 38 | F | 10-01-21 | 30-01-21 | 15-02-21 |
| 13 | 32-MT | 47 | F | 13-01-21 |  | 15-02-21 |
| 14 | 36-DDA | 35 | F | 11-01-21 |  | 29-01-21 |
| 15 | 47-SA | 43 | M | 13-01-21 | 03-02-21 | 19-02-21 |
| 16 | 48-PF | 27 | F | 13-01-21 | 03-02-21 | 19-02-21 |
| 17 | 50-AC | 32 | M | 13-01-21 | 03-02-21 | 16-02-21 |
| 18 | 59-GA | 44 | M |  | 05-02-21 | 19-02-21 |
| 19 | 64-VDD | 25 | F | 03-01-21 | 24-01-21 | 08-02-21 |

**Supplementary Table S3. HLA genotype data for patient donors**

| **S. No.** | **Patient ID** | **HLA-A** | | **HLA-B** | | **HLA-C** | |
| --- | --- | --- | --- | --- | --- | --- | --- |
| 1 | PTVACC-01 | 02:01:01:01 | 24:02:01:05 | 15:01:01:01 | 18:01:01 | 03:04:01 | 07:01:01 |
| 2 | PTVACC-02 | 01:01:01:01 | 32:01:01:01 | 08:01:01 | 44:02:01 | 06:02:01 | 07:01:01:06 |
| 3 | PTVACC-03 | 01:01:01:01 | 01:01:01:01 | 07:02:01 | 08:09 | 07:01:01 | 07:02:01 |
| 4 | PTVACC-04 | 03:01:01:01 | 25:01:01:01 | 07:02:01 | 18:01:01 | 07:02:01 | 12:03:01 |
| 5 | PTVACC-05 | 03:01:01:01 | 25:01:01:01 | 07:02:01 | 18:01:01 | 07:02:01 | 12:03:01 |
| 6 | PTVACC-06 | 02:01:01:01 | 11:01:01:01 | 07:02:01 | 39:24:01 | 07:01:01 | 07:02:01 |
| 7 | PTVACC-07 | 01:01:01:01 | 03:01:01:01 | 07:02:01 | 35:03:01 | 04:01:01 | 07:10 |
| 8 | PTVACC-08 | 24:02:01:01 | 24:02:01:01 | 07:02:01 | 40:01:02 | 03:04:01 | 07:02:01 |
| 9 | PTVACC-09 | 02:01:01:01 | 02:01:01:01 | 13:02:01 | 27:05:02 | 01:02:01 | 06:02:01 |
| 10 | PTVACC-10 | 01:01:01:01 | 33:01:01:01 | 08:01:01 | 14:02:01:01 | 07:01:01 | 08:02:01 |
| 11 | PTVACC-12 | 02:01:01:01 | 03:01:01:01 | 15:01:01:01 | 49:01:01 | 03:04:01 | 07:01:01 |
| 12 | PTVACC-13 | 01:01:01:01 | 31:01:02:01 | 08:01:01 | 40:01:02 | 03:04:01 | 07:01:01 |
| 13 | PTVACC-14 | 01:01:01:01 | 31:01:02:01 | 08:01:01 | 37:01:01:01 | 06:02:01 | 07:01:01 |
| 14 | PTVACC-16 | 01:01:01:01 | 11:01:01:01 | 08:01:01 | 27:05:02 | 02:02:02 | 07:01:01 |
| 15 | PTVACC-17 | 26:01:01:01 | 31:01:02:01 | 07:02:01 | 14:01:01:01 | 07:02:01 | 08:02:01:02 |
| 16 | PTVACC-18 | 26:01:01:01 | 29:02:01:01 | 07:02:01 | 44:03:01 | 07:02:01 | 16:01:01:01 |
| 17 | PTVACC-19 | 03:01:01:01 | 03:01:01:01 | 07:02:01 | 44:02:01 | 05:01:01 | 07:02:01 |
| 18 | PTVACC-20 | 01:01:01:01 | 03:01:01:01 | 08:01:01 | 35:01:01 | 04:01:01 | 07:01:01 |
| 19 | PTVACC-21 | 02:01:01:01 | 24:03:01:01 | 15:01:01:01 | 38:01:01 | 03:04:01 | 12:03:01 |
| 20 | PTVACC-23 | 24:02:01:01 | 26:01:01:01 | 08:01:01 | 39:06:02:01 | 07:01:01 | 07:02:01 |
| 21 | PTVACC-25 | 24:02:01:01 | 29:02:01:01 | 15:01:01:01 | 44:03:01 | 03:03:01 | 16:01:01:01 |
| 22 | PTVACC-26 | 02:01:01:01 | 02:01:01:01 | 15:01:01:01 | 40:01:02 | 03:03:01 | 03:04:01 |
| 23 | PTVACC-28 | 02:01:01:01 | 02:05:01:01 | 50:01:01:06 | 51:01:01 | 06:02:01:02 | 15:02:01 |
| 24 | PTVACC-29 | 03:01:01:01 | 30:02:01:01 | 07:02:01 | 18:01:01 | 05:01:01 | 07:02:01 |
| 25 | PTVACC-30 | 02:01:01:01 | 31:01:02:01 | 40:01:02 | 44:02:01 | 03:04:01 | 07:04:01 |
| 26 | PTVACC-31 | 02:01:01:01 | 25:01:01:01 | 39:01:01 | 50:01:01:01 | 06:02:01:02 | 07:02:01 |
| 27 | PTVACC-32 | 01:01:01:01 | 24:02:01:01 | 07:02:01 | 08:01:01 | 07:01:01 | 07:02:01 |
| 28 | PTVACC-33 | 02:01:01:01 | 02:01:01:01 | 37:01:01:01 | 57:01:01 | 06:02:01 | 06:02:01 |

**Supplementary Table S4. HLA genotype data for healthy donors**

| **S. No.** | **Healthy donor ID** | **HLA-A** | |
| --- | --- | --- | --- |
| 1 | 3-GS | 01:01 | 30:01 |
| 2 | 4-FM | 02:01 | 24:02 |
| 3 | 7-RDF | 02:01 | 24:02 |
| 4 | 9-PM | 01:01 | 03:01 |
| 5 | 10-PA | 01:01 | 23:01P |
| 6 | 12-BV | 03:01 | 24:02 |
| 7 | 13-GB | 01:01 | 32:01 |
| 8 | 15-CR | 02:01 | 11:01 |
| 9 | 21-AI | 01:01 | 02:01 |
| 10 | 24-DR | 02:01 | 11:01 |
| 11 | 25-CE | 02:01 | 11:01 |
| 12 | 27-ACP | 01:01 | 69:01 |
| 13 | 32-MT | 03:01 | 24:02 |
| 14 | 36-DDA | 03:01 | 68:01 |
| 15 | 47-SA | 02:01 | 24:02 |
| 16 | 48-PF | 01:01 | 02:01 |
| 17 | 50-AC | 03:01 | 68:02 |
| 18 | 59-GA | 01:01 | 24:02 |
| 19 | 64-VDD | 02:01 | 03:01 |

**Supplementary Table S6. CEF peptide library**

| **Number** | **HLA** | **Virus** | **Peptide sequence** |
| --- | --- | --- | --- |
| 1 | HLA-A01:01 | CMV | YSEHPTFTSQY |
| 2 | HLA-A01:01 | CMV | VTEHDTLLY |
| 3 | HLA-A01:01 | Influenza | VSDGGPNLY |
| 4 | HLA-A01:01 | Influenza | CTELKLSDY |
| 5 | HLA-A01:01 | Influenza | TFEFTSFFY |
| 6 | HLA-A01:01 | Influenza | AEKPKFLPDLY |
| 7 | HLA-A02:01 | Influenza | FMYSDFHFI |
| 8 | HLA-A02:01 | Influenza | AIMDKNIML |
| 9 | HLA-A02:01 | Influenza | AIMDKNIIL |
| 10 | HLA-A02:01 | Influenza | ILGFVFLTV |
| 11 | HLA-A02:01 | Influenza | MMMGMFNML |
| 12 | HLA-A02:01 | Influenza | FNMLSTVLGV |
| 13 | HLA-A03:01 | Influenza | SIIPSGPLK |
| 14 | HLA-A03:01 | Influenza | RMVLASTTAK |
| 15 | HLA-A03:01 | Influenza | RVLSFIKGTK |
| 16 | HLA-A03:01 | Influenza | RLEDVFAGK |
| 17 | HLA-A03:01 | Influenza | KSMREEYRK |
| 18 | HLA-A11:01 | EBV | AVFDRKSDAK |
| 19 | HLA-A11:01 | CMV | GPISGHVLK |
| 20 | HLA-A11:01 | Influenza | SIIPSGPLK |
| 21 | HLA-A11:01 | Influenza | RMVLASTTAK |
| 22 | HLA-A11:01 | Influenza | RVLSFIKGTK |
| 23 | HLA-A11:01 | Influenza | RLEDVFAGK |
| 24 | HLA-A11:01 | Influenza | KSMREEYRK |
| 25 | HLA-A24:02 | EBV | RYSIFFDY |
| 26 | HLA-A24:02 | EBV | TYGPVFMCL |
| 27 | HLA-A24:02 | EBV | DYCNVLNKEF |
| 28 | HLA-A24:02 | CMV | AYAQKIFKIL |
| 29 | HLA-A24:02 | Influenza | FMYSDFHFI |
| 30 | HLA-A24:02 | Influenza | SWPDGAELPF |
| 31 | HLA-A24:02 | Influenza | ITFMQALQLL |
| 32 | HLA-A24:02 | Influenza | VETPIRNEW |
| 33 | HLA-B07:02 | CMV | TPRVTGGGAM |
| 34 | HLA-B07:02 | CMV | RPHERNGFTV |
| 35 | HLA-B07:02 | EBV | RPPIFIRLL |
| 36 | HLA-B07:02 | Influenza | AIMDKNIML |
| 37 | HLA-B07:02 | Influenza | AIMDKNIIL |
| 38 | HLA-B07:02 | Influenza | SWPDGAELPF |
| 39 | HLA-B07:02 | Influenza | TTYQRTRAL |
| 40 | HLA-B07:02 | Influenza | LPFDKPTIM |
| 41 | HLA-B07:02 | Influenza | LPFDKTTVM |
| 42 | HLA-B07:02 | Influenza | LPFEKSTVM |
| 43 | HLA-B07:02 | Influenza | LPFDKSTIM |
| 44 | HLA-B07:02 | Influenza | LPFERSTIM |
| 45 | HLA-B07:02 | Influenza | LPFERATIM |
| 46 | HLA-B07:02 | Influenza | QPEWFRNVL |
| 47 | HLA-B07:02 | Influenza | SPIVPSFDM |
| 48 | HLA-B08:01 | Influenza | ELRSRYWAI |
| 49 | HLA-B08:01 | EBV | RAKFKQLL |
| 50 | HLA-B08:01 | CMV | ELRRKMMYM |
| 51 | HLA-B08:01 | EBV | QAKWRLQTL |
| 52 | HLA-B08:01 | EBV | FLRGRAYGL |
| 53 | HLA-B08:01 | Influenza | AIMDKNIML |
| 54 | HLA-B08:01 | Influenza | AIMDKNIIL |
| 55 | HLA-B08:01 | Influenza | MMMGMFNML |
| 56 | HLA-B08:01 | Influenza | TTYQRTRAL |
| 57 | HLA-B08:01 | Influenza | LPFDKPTIM |
| 58 | HLA-B08:01 | Influenza | LPFDKTTVM |
| 59 | HLA-B08:01 | Influenza | LPFEKSTVM |
| 60 | HLA-B08:01 | Influenza | LPFDKSTIM |
| 61 | HLA-B08:01 | Influenza | LPFERSTIM |
| 62 | HLA-B08:01 | Influenza | LPFERATIM |
| 63 | HLA-B15:01 | EBV | QNGALAINTF |
| 64 | HLA-B15:01 | EBV | LEKARGSTY |
| 65 | HLA-B15:01 | Influenza | TQIQTRRSF |
| 66 | HLA-B15:01 | Influenza | KMARLGKGY |
| 67 | HLA-B15:01 | EBV | GQGGSPTAM |

**Supplementary Table S7. Antibody panels**

| **Antibody phenotype panel used with DNA-barcoded pMHC multimers staining** | | | | | |
| --- | --- | --- | --- | --- | --- |
| **Antibody** | **Conjugate** | **Clone** | **Dilution** | **Provider** | **Catalogue ID** |
| CD3 | BV786 | SK7 | 1/20 | BD Biosciences | 563800 |
| CD4 | BV650 | SK3 | 1/40 | BD Biosciences | 563875 |
| CD8 | BV480 | RPA-T8 | 1/50 | BD Biosciences | 566121 |
| CD45RA | BV711 | HI100 | 1/40 | BD Biosciences | 563733 |
| CCR7 | FITC | G043H7 | 1/20 | Biolegend | 353216 |
| CD27 | BV605 | O323 | 1/40 | Biolegend | 302830 |
| CD38 | BUV737 | HB7 | 1/160 | BD Biosciences | 612824 |
| CD39 | PE-CF594 | Tu66 | 1/40 | BD Biosciences | 563678 |
| CD69 | BUV395 | FN50 | 1/20 | BD Biosciences | 564364 |
| CD137 | PE-Cy5 | 4B4-1 | 1/40 | BD Biosciences | 551137 |
| HLA-DR | APC-R700 | G46-6 | 1/160 | BD Biosciences | 565127 |
| PD1 | BV421 | EH12.1 | 1/33 | BioLegend | 562516 |
| Live-Dead marker | APC-Cy7 | - | 1/1000 | Invitrogen | L34976 |

| **Antibody phenotype panel used with pMHC tetramer staining** | | | | | |
| --- | --- | --- | --- | --- | --- |
| **Antibody** | **Conjugate** | **Clone** | **Dilution** | **Provider** | **Catalogue ID** |
| CD8 | BV480 | RPA-T8 | 1/50 | BD Biosciences | 566121 |
| Live-Dead marker | APC-Cy7 | - | 1/1000 | Invitrogen | L34976 |
| Dump channel antibodies | | | | | |
| CD4 | FITC | SK3 | 1/80 | BD Biosciences | 345768 |
| CD14 | FITC | MφP9 | 1/32 | BD Biosciences | 345784 |
| CD19 | FITC | 4G7 | 1/16 | BD Biosciences | 345776 |
| CD40 | FITC | LOB7/6 | 1/40 | Serotech | MCA1590F |
| CD16 | FITC | NKP15 | 1/64 | BD Biosciences | 335035 |

| **Antibodies used for intracellular cytokine staining** | | | | | |
| --- | --- | --- | --- | --- | --- |
| **Antibody** | **Conjugate** | **Clone** | **Dilution** | **Provider** | **Catalogue ID** |
| Surface marker antibodies | | | | | |
| CD3 | FITC | SK7 | 1/20 | BD Biosciences | 345764 |
| CD4 | BUV395 | SK3 | 1/300 | BD Biosciences | 563550 |
| CD8 | BV480 | RPA-T8 | 1/50 | BD Biosciences | 566121 |
| Live-Dead marker | APC-Cy7 | - | 1/1000 | Invitrogen | L34976 |
| Intracellular antibodies | | | | | |
| TNFα | PE-Cy7 | - | 1/20 | BioLegend | 502930 |
| IFNγ | APC | - | 1/20 | BD Biosciences | 341117 |

**Supplementary Table S9. List of unique SARS-CoV-2 spike-specific responses identified in HM patients pre- and post-vaccination**

| **No.** | **Peptide** | **HLA** | **n*** |
| --- | --- | --- | --- |
| 1 | FCNDPFLGVY | A01:01 | 1 |
| 2 | LTDEMIAQY | A01:01 | 1 |
| 3 | PLLTDEMIAQY | A01:01 | 1 |
| 4 | QTLLALHRSY | A01:01 | 2 |
| 5 | SGWTAGAAAYY | A01:01 | 5 |
| 6 | SSANNCTFEY | A01:01 | 1 |
| 7 | STECSNLLL | A01:01 | 7 |
| 8 | TDEMIAQY | A01:01 | 1 |
| 9 | TTEILPVSM | A01:01 | 1 |
| 10 | WTAGAAAYY | A01:01 | 1 |
| 11 | ALNTLVKQL | A02:01 | 2 |
| 12 | HLMSFPQSA | A02:01 | 1 |
| 13 | NLNESLIDL | A02:01 | 1 |
| 14 | RLDKVEAEVQI | A02:01 | 1 |
| 15 | RLITGRLQSL | A02:01 | 2 |
| 16 | RLNEVAKNL | A02:01 | 4 |
| 17 | VLSFELLHA | A02:01 | 1 |
| 18 | VLYQDVNCTEV | A02:01 | 2 |
| 19 | VTWFHAIHV | A02:01 | 3 |
| 20 | YLQPRTFLL | A02:01 | 16 |
| 21 | YQDVNCTEV | A02:01 | 1 |
| 22 | GVYFASTEK | A03:01 | 2 |
| 23 | GVYYPDKVFR | A03:01 | 1 |
| 24 | KCYGVSPTK | A03:01 | 1 |
| 25 | EYVSQPFLM | A24:02 | 1 |
| 26 | GNYNYLYRLF | A24:02 | 2 |
| 27 | GYLQPRTF | A24:02 | 1 |
| 28 | GYQPYRVVV | A24:02 | 1 |
| 29 | IYKTPPIKDF | A24:02 | 1 |
| 30 | LFLPFFSNVTW | A24:02 | 1 |
| 31 | NYNYLYRLF | A24:02 | 12 |
| 32 | PFAMQMAYRF | A24:02 | 1 |
| 33 | PFGEVFNATRF | A24:02 | 1 |
| 34 | QPYRVVVLSF | A24:02 | 6 |
| 35 | QYIKWPWYI | A24:02 | 2 |
| 36 | RVYSSANNCTF | A24:02 | 1 |
| 37 | SFKEELDKYF | A24:02 | 1 |
| 38 | TQDLFLPFF | A24:02 | 1 |
| 39 | TVYDPLQPEL | A24:02 | 5 |
| 40 | VFVSNGTHW | A24:02 | 1 |
| 41 | VGYLQPRTFL | A24:02 | 1 |
| 42 | VYSTGSNVF | A24:02 | 1 |
| 43 | YFPLQSYGF | A24:02 | 1 |
| 44 | YLQPRTFLL | A24:02 | 1 |
| 45 | YYHKNNKSW | A24:02 | 1 |
| 46 | YYVGYLQPRTF | A24:02 | 1 |
| 47 | APHGVVFL | B07:02 | 1 |
| 48 | SPRRARSV | B07:02 | 2 |
| 49 | DLPQGFSAL | B08:01 | 1 |
| 50 | FKNLREFVF | B08:01 | 1 |
| 51 | LITGRLQSL | B08:01 | 1 |
| 52 | LPQGFSAL | B08:01 | 1 |
| 53 | QPYRVVVL | B08:01 | 1 |
| 54 | VAKNLNESL | B08:01 | 1 |
| 55 | CVADYSVLY | B15:01 | 4 |
| 56 | GQTGKIADY | B15:01 | 2 |
| 57 | GQTGKIADYNY | B15:01 | 1 |
| 58 | LPFNDGVYF | B35:01 | 1 |
| 59 | VLPFNDGVYF | B35:01 | 1 |

n*= number of T-cell responses over all time points

**Supplementary Table S10: List of identified SARS-CoV-2 Spike and CEF-derived epitopes in each healthy donor (Donors detected for a SARS-CoV-2- or CEF-specific CD8+ T-cells)**

| **Time point** | **Sample** | **SARS-CoV-2** | **CEF** | **HLA** | **Peptide** | **Est. frequency** | **Log fold**  **change** | **p** |
| --- | --- | --- | --- | --- | --- | --- | --- | --- |
| **TP2** | 4-FM | Spike |  | B07:02 | APHGVVFLHV | 0.00438965 | 2.774271135 | 4.21449E-07 |
|  | 7-RDF | Spike |  | A24:02 | NYNYLYRLF | 0.00073313 | 2.62053158 | 7.96152E-06 |
|  |  |  | EBV | B08:01 | RAKFKQLL | 1.302277433 | 2.220737918 | 0.000347742 |
|  | 10-PA | Spike |  | A01:01 | CNDPFLGVYY | 0.000294435 | 2.476331911 | 4.77679E-05 |
|  |  |  | CMV | A01:01 | VTEHDTLLY | 0.066164466 | 3.453303108 | 5.40338E-10 |
|  |  |  | CMV | A01:01 | YSEHPTFTSQY | 0.041188475 | 3.083039593 | 8.35565E-08 |
|  | 12-BV | Spike |  | A24:02 | NYNYLYRLF | 0.000279518 | 2.658187479 | 6.50962E-06 |
|  |  | Spike |  | B07:02 | KPSKRSFIEDL | 0.00079386 | 2.280902663 | 0.000120398 |
|  | 13-GB |  | CMV | A01:01 | VTEHDTLLY | 0.472221287 | 5.224792289 | 5.50166E-24 |
|  |  |  | CMV | B07:02 | TPRVTGGGAM | 1.402605853 | 6.188242637 | 5.68816E-32 |
|  |  |  | EBV | B08:01 | RAKFKQLL | 0.14739609 | 3.617217243 | 3.95271E-12 |
|  | 15-CR | Spike |  | B08:01 | VFQTRAGCL | 0.001879509 | 4.604412646 | 3.63587E-19 |
|  |  |  | EBV | A11:01 | AVFDRKSDAK | 0.015641379 | 2.40040148 | 9.63192E-05 |
|  | 21-AI |  | CMV | A01:01 | VTEHDTLLY | 0.046867729 | 2.855837105 | 4.51807E-07 |
|  | 24-DR | Spike |  | A02:01 | YLQPRTFLL | 0.003518624 | 2.661312193 | 1.7102E-06 |
|  |  |  | EBV | A11:01 | AVFDRKSDAK | 0.100966921 | 2.690882902 | 7.73563E-06 |
|  | 25-CE | Spike |  | A02:01 | YLQPRTFLL | 0.003356941 | 3.557378751 | 8.99132E-12 |
|  |  | Spike |  | B07:02 | APGQTGKIA | 0.018466541 | 5.886907856 | 1.6134E-29 |
|  | 27-ACP |  | CMV | A01:01 | VTEHDTLLY | 0.933285519 | 7.422775844 | 2.0529E-42 |
|  | 32-MT | Spike |  | A03:01 | KCYGVSPTK | 0.001940778 | 3.047260776 | 4.49303E-08 |
|  |  | Spike |  | A24:02 | NYNYLYRLF | 0.002133651 | 2.583660931 | 1.17714E-05 |
|  |  |  | EBV | B08:01 | RAKFKQLL | 0.015261876 | 4.259625859 | 3.6431E-16 |
|  |  |  | EBV | B08:01 | FLRGRAYGL | 0.010499391 | 2.281967002 | 8.7806E-05 |
|  | 50-AC | Spike |  | B07:02 | VVNQNAQAL | 0.002737023 | 2.726455031 | 8.86024E-07 |
|  |  | Spike |  | B35:01 | HADQLTPTW | 0.002822398 | 2.259543364 | 0.000273509 |
|  | 59-GA |  | Influenza | B08:01 | TTYQRTRAL | 0.049391289 | 2.223693611 | 0.000507732 |
|  | 64-VDD | Spike |  | A02:01 | YLQPRTFLL | 0.00284029 | 2.245876745 | 0.000176079 |

**Supplementary Table S11. SARS-CoV-2 Spike T cell epitopes previously reported from natural infection**.

| **HLA** | **Peptide** | **Reference** |
| --- | --- | --- |
| **A01:01** | LLTDEMIAQY | [Saini et al. 2021](https://www.science.org/doi/10.1126/sciimmunol.abf7550) |
|  | LTDEMIAQY | [Schulien et al. 2021](https://www.nature.com/articles/s41591-020-01143-2); [Nelde et al. 2021](https://www.nature.com/articles/s41590-020-00808-x); [Kared et al. 2021](https://pubmed.ncbi.nlm.nih.gov/33427749/) |
|  | **WTAGAAAYY**^a^ | [Wagner et al. 2022](https://www.cell.com/cell-reports/fulltext/S2211-1247(21)01718-6) |
|  | YTNSFTRGVY | [Tarke et al. 2021](https://www.sciencedirect.com/science/article/pii/S266637912100015X) |
|  | YTNSFTRGVY | [Tarke et al. 2021](https://www.sciencedirect.com/science/article/pii/S266637912100015X) |
| **A02:01** | ALNTLVKQL | [Shomuradova et al. 2020](https://www.cell.com/immunity/fulltext/S1074-7613(20)30469-6) |
|  | FIAGLIAIV | [Poran et al. 2020](https://genomemedicine.biomedcentral.com/articles/10.1186/s13073-020-00767-w); [Rha et al. 2021](https://www.cell.com/immunity/fulltext/S1074-7613(20)30509-4); [Shomuradova et al. 2020](https://www.cell.com/immunity/fulltext/S1074-7613(20)30469-6); [Saini et al. 2021](https://www.science.org/doi/10.1126/sciimmunol.abf7550) |
|  | FLPFFSNV | [Saini et al. 2021](https://www.science.org/doi/10.1126/sciimmunol.abf7550?url_ver=Z39.88-2003&rfr_id=ori:rid:crossref.org&rfr_dat=cr_pub%20%200pubmed) |
|  | GLTVLPPLL | [Tarke et al. 2021](https://www.sciencedirect.com/science/article/pii/S266637912100015X); [Poran et al. 2020](https://genomemedicine.biomedcentral.com/articles/10.1186/s13073-020-00767-w) |
|  | HLMSFPQSA | [Tarke et al. 2021](https://www.sciencedirect.com/science/article/pii/S266637912100015X) |
|  | KIADYNYKL | [Chen et al. 2020](https://www.ncbi.nlm.nih.gov/pmc/articles/PMC7812294/); [Shomuradova et al. 2020](https://www.cell.com/immunity/fulltext/S1074-7613(20)30469-6) |
|  | KIADYNYKL | [Shomuradova et al. 2020](https://www.cell.com/immunity/fulltext/S1074-7613(20)30469-6) |
|  | KLNDLCFTNV | [Poran et al. 2020](https://genomemedicine.biomedcentral.com/articles/10.1186/s13073-020-00767-w) |
|  | KLPDDFTGCV | [Shomuradova et al. 2020](https://www.cell.com/immunity/fulltext/S1074-7613(20)30469-6) |
|  | KLPDDFTGCV | [Shomuradova et al. 2020](https://www.cell.com/immunity/fulltext/S1074-7613(20)30469-6) |
|  | LITGRLQSL | [Shomuradova et al. 2020](https://www.cell.com/immunity/fulltext/S1074-7613(20)30469-6) |
|  | LLFNKVTLA | [Shomuradova et al. 2020](https://www.cell.com/immunity/fulltext/S1074-7613(20)30469-6) |
|  | **RLNEVAKNL**^a^ | [Shomuradova et al. 2020](https://www.cell.com/immunity/fulltext/S1074-7613(20)30469-6) |
|  | RLQSLQTYV | [Tarke et al. 2021](https://www.sciencedirect.com/science/article/pii/S266637912100015X); [Shomuradova et al. 2020](https://www.cell.com/immunity/fulltext/S1074-7613(20)30469-6); [Poran et al. 2020](https://genomemedicine.biomedcentral.com/articles/10.1186/s13073-020-00767-w) |
|  | SIIAYTMSL | [Tarke et al. 2021](https://www.sciencedirect.com/science/article/pii/S266637912100015X) |
|  | TLDSKTQSL | [Tarke et al. 2021](https://www.sciencedirect.com/science/article/pii/S266637912100015X); [Sekine et al. 2020](https://www.cell.com/cell/fulltext/S0092-8674(20)31008-4) |
|  | VLNDILSRL | [Habel et al. 2020](https://www.pnas.org/doi/10.1073/pnas.2015486117); [Shomuradova et al. 2020](https://www.cell.com/immunity/fulltext/S1074-7613(20)30469-6); [Saini et al. 2021](https://www.science.org/doi/10.1126/sciimmunol.abf7550) |
|  | VVFLHVTYV | [Kared et al. 2021](https://pubmed.ncbi.nlm.nih.gov/33427749/); [Tarke et al. 2021](https://www.sciencedirect.com/science/article/pii/S266637912100015X); [Saini et al. 2021](https://www.science.org/doi/10.1126/sciimmunol.abf7550) |
|  | **YLQPRTFLL**^a^ | [Ferretti et al. 2020](https://www.ncbi.nlm.nih.gov/pmc/articles/PMC7574860/); [Tarke et al. 2021](https://www.sciencedirect.com/science/article/pii/S266637912100015X); [Shomuradova et al. 2020](https://www.cell.com/immunity/fulltext/S1074-7613(20)30469-6); [Rha et al. 2021](https://www.cell.com/immunity/fulltext/S1074-7613(20)30509-4); [Sekine et al. 2020](https://www.cell.com/cell/fulltext/S0092-8674(20)31008-4); [Habel et al. 2020](https://www.pnas.org/doi/10.1073/pnas.2015486117); [Kared et al. 2021](https://pubmed.ncbi.nlm.nih.gov/33427749/) |
| **A03:01** | ALDPLSETK | [Tarke et al. 2021](https://www.sciencedirect.com/science/article/pii/S266637912100015X) |
|  | EILPVSMTK | [Tarke et al. 2021](https://www.sciencedirect.com/science/article/pii/S266637912100015X) |
|  | GVYFASTEK | [Kared et al. 2021](https://pubmed.ncbi.nlm.nih.gov/33427749/) |
|  | GVYYHKNNK | [Tarke et al. 2021](https://www.sciencedirect.com/science/article/pii/S266637912100015X) |
|  | GVYYPDKVFR | [Tarke et al. 2021](https://www.sciencedirect.com/science/article/pii/S266637912100015X) |
|  | KCYGVSPTK | [Ferretti et al. 2020](https://www.ncbi.nlm.nih.gov/pmc/articles/PMC7574860/); [Tarke et al. 2021](https://www.sciencedirect.com/science/article/pii/S266637912100015X); [Saini et al. 2021](https://www.science.org/doi/10.1126/sciimmunol.abf7550) |
|  | KVFRSSVLH | [Tarke et al. 2021](https://www.sciencedirect.com/science/article/pii/S266637912100015X) |
|  | RASANLAATK | [Tarke et al. 2021](https://www.sciencedirect.com/science/article/pii/S266637912100015X) |
|  | RLFRKSNLK | [Tarke et al. 2021](https://www.sciencedirect.com/science/article/pii/S266637912100015X) |
|  | SVYAWNRKR | [Tarke et al. 2021](https://www.sciencedirect.com/science/article/pii/S266637912100015X) |
|  | TLADAGFIK | [Tarke et al. 2021](https://www.sciencedirect.com/science/article/pii/S266637912100015X) |
|  | TVYDPLQPELDSFK | [Tarke et al. 2021](https://www.sciencedirect.com/science/article/pii/S266637912100015X) |
|  | VTYVPAQEK | [Tarke et al. 2021](https://www.sciencedirect.com/science/article/pii/S266637912100015X) |
| **A11:01** | GTHWFVTQR | [Kared et al. 2021](https://pubmed.ncbi.nlm.nih.gov/33427749/) |
|  | GVYFASTEK | [Kared et al. 2021](https://pubmed.ncbi.nlm.nih.gov/33427749/) |
|  | RLFRKSNLK | [Kared et al. 2021](https://pubmed.ncbi.nlm.nih.gov/33427749/) |
| **A24:02** | AYSNNSIAI | [Tarke et al. 2021](https://www.sciencedirect.com/science/article/pii/S266637912100015X) |
|  | EYVSQPFLM | [Tarke et al. 2021](https://www.sciencedirect.com/science/article/pii/S266637912100015X) |
|  | GYLQPRTFLL | [Tarke et al. 2021](https://www.sciencedirect.com/science/article/pii/S266637912100015X) |
|  | HWFVTQRNF | [Tarke et al. 2021](https://www.sciencedirect.com/science/article/pii/S266637912100015X) |
|  | IYQTSNFRV | [Tarke et al. 2021](https://www.sciencedirect.com/science/article/pii/S266637912100015X) |
|  | **NYNYLYRLF**^a^ | [Tarke et al. 2021](https://www.sciencedirect.com/science/article/pii/S266637912100015X); [Kared et al. 2021](https://pubmed.ncbi.nlm.nih.gov/33427749/) |
|  | **QYIKWPWYI**^a^ | [Ferretti et al. 2020](https://www.ncbi.nlm.nih.gov/pmc/articles/PMC7574860/); [Tarke et al. 2021](https://www.sciencedirect.com/science/article/pii/S266637912100015X); [Nelde et al. 2021](https://www.nature.com/articles/s41590-020-00808-x); [Kared et al. 2021](https://pubmed.ncbi.nlm.nih.gov/33427749/) |
|  | RFDNPVLPF | [Tarke et al. 2021](https://www.sciencedirect.com/science/article/pii/S266637912100015X); [Kared et al. 2021](https://pubmed.ncbi.nlm.nih.gov/33427749/) |
|  | RFPNITNLCPF | [Tarke et al. 2021](https://www.sciencedirect.com/science/article/pii/S266637912100015X) |
|  | RVYSTGSNVF | [Tarke et al. 2021](https://www.sciencedirect.com/science/article/pii/S266637912100015X) |
|  | RVYSTGSNVF | [Tarke et al. 2021](https://www.sciencedirect.com/science/article/pii/S266637912100015X) |
|  | SFPQSAPHGVVF | [Tarke et al. 2021](https://www.sciencedirect.com/science/article/pii/S266637912100015X) |
|  | **TQDLFLPFF**^a^ | [Saini et al. 2021](https://www.science.org/doi/10.1126/sciimmunol.abf7550) |
|  | TYVPAQEKNFT | [Saini et al. 2021](https://www.science.org/doi/10.1126/sciimmunol.abf7550) |
|  | VFKNIDGYF | [Tarke et al. 2021](https://www.sciencedirect.com/science/article/pii/S266637912100015X) |
|  | VFVSNGTHWF | [Tarke et al. 2021](https://www.sciencedirect.com/science/article/pii/S266637912100015X) |
|  | VYDPLQPELDSF | [Tarke et al. 2021](https://www.sciencedirect.com/science/article/pii/S266637912100015X) |
|  | VYSSANNCTF | [Tarke et al. 2021](https://www.sciencedirect.com/science/article/pii/S266637912100015X) |
|  | VYYPDKVF | [Tarke et al. 2021](https://www.sciencedirect.com/science/article/pii/S266637912100015X) |
|  | YYHKNNKSW | [Tarke et al. 2021](https://www.sciencedirect.com/science/article/pii/S266637912100015X) |
|  | YYVGYLQPRTF | [Tarke et al. 2021](https://www.sciencedirect.com/science/article/pii/S266637912100015X) |
| **B07:02** | **APHGVVFL**^a^ | [Kared et al. 2021](https://pubmed.ncbi.nlm.nih.gov/33427749/) |
|  | EPVLKGVKL | [Tarke et al. 2021](https://www.sciencedirect.com/science/article/pii/S266637912100015X) |
|  | EPVLKGVKL | [Tarke et al. 2021](https://www.sciencedirect.com/science/article/pii/S266637912100015X) |
|  | FPQSAPHGV | [Tarke et al. 2021](https://www.sciencedirect.com/science/article/pii/S266637912100015X) |
|  | IPTNFTISV | [Tarke et al. 2021](https://www.sciencedirect.com/science/article/pii/S266637912100015X) |
|  | KPFERDISTEI | [Tarke et al. 2021](https://www.sciencedirect.com/science/article/pii/S266637912100015X) |
|  | LPFNDGVYF | [Tarke et al. 2021](https://www.sciencedirect.com/science/article/pii/S266637912100015X) |
|  | LPIGINITRF | [Tarke et al. 2021](https://www.sciencedirect.com/science/article/pii/S266637912100015X) |
|  | LPPAYTNSF | [Tarke et al. 2021](https://www.sciencedirect.com/science/article/pii/S266637912100015X) |
|  | LPQGFSAL | [Tarke et al. 2021](https://www.sciencedirect.com/science/article/pii/S266637912100015X) |
|  | MIAQYTSAL | [Saini et al. 2021](https://www.science.org/doi/10.1126/sciimmunol.abf7550) |
|  | QPTESIVRF | [Tarke et al. 2021](https://www.sciencedirect.com/science/article/pii/S266637912100015X) |
|  | QPYRVVVL | [Tarke et al. 2021](https://www.sciencedirect.com/science/article/pii/S266637912100015X) |
|  | QPYRVVVLSF | [Tarke et al. 2021](https://www.sciencedirect.com/science/article/pii/S266637912100015X) |
|  | SPRRARSVA | [Schulien et al. 2021](https://www.nature.com/articles/s41591-020-01143-2) |
|  | SPRRARSVA | [Schulien et al. 2021](https://www.nature.com/articles/s41591-020-01143-2) |
|  | TPCSFGGVSV | [Tarke et al. 2021](https://www.sciencedirect.com/science/article/pii/S266637912100015X) |
|  | TPCSFGGVSV | [Tarke et al. 2021](https://www.sciencedirect.com/science/article/pii/S266637912100015X) |
|  | TPINLVRDL | [Tarke et al. 2021](https://www.sciencedirect.com/science/article/pii/S266637912100015X) |
|  | TPINLVRDL | [Tarke et al. 2021](https://www.sciencedirect.com/science/article/pii/S266637912100015X) |
| **B08:01** | INITRFQTL | [Tarke et al. 2021](https://www.sciencedirect.com/science/article/pii/S266637912100015X) |
|  | KIYSKHTPI | [Tarke et al. 2021](https://www.sciencedirect.com/science/article/pii/S266637912100015X) |
|  | LPQGFSAL | [Tarke et al. 2021](https://www.sciencedirect.com/science/article/pii/S266637912100015X); [Saini et al. 2021](https://www.science.org/doi/10.1126/sciimmunol.abf7550) |
|  | MIAQYTSAL | [Tarke et al. 2021](https://www.sciencedirect.com/science/article/pii/S266637912100015X) |
|  | MIAQYTSAL | [Tarke et al. 2021](https://www.sciencedirect.com/science/article/pii/S266637912100015X) |
|  | NITRFQTL | [Tarke et al. 2021](https://www.sciencedirect.com/science/article/pii/S266637912100015X) |
|  | QPYRVVVL | [Tarke et al. 2021](https://www.sciencedirect.com/science/article/pii/S266637912100015X) |
|  | SIIAYTMSL | [Tarke et al. 2021](https://www.sciencedirect.com/science/article/pii/S266637912100015X) |
|  | SPRRARSV | [Tarke et al. 2021](https://www.sciencedirect.com/science/article/pii/S266637912100015X) |
|  | TLDSKTQSL | [Tarke et al. 2021](https://www.sciencedirect.com/science/article/pii/S266637912100015X) |
|  | YLQPRTFLL | [Tarke et al. 2021](https://www.sciencedirect.com/science/article/pii/S266637912100015X) |
| **B15:01** | CVADYSVLY | [Saini et al. 2021](https://www.science.org/doi/10.1126/sciimmunol.abf7550) |
|  | LVKNKCVNF | [Saini et al. 2021](https://www.science.org/doi/10.1126/sciimmunol.abf7550) |
|  | RLQSLQTY | [Tarke et al. 2021](https://www.sciencedirect.com/science/article/pii/S266637912100015X) |
|  | RVYSTGSNVF | [Tarke et al. 2021](https://www.sciencedirect.com/science/article/pii/S266637912100015X) |
|  | VASQSIIAY | [Saini et al. 2021](https://www.science.org/doi/10.1126/sciimmunol.abf7550) |
| **B35:01** | FAMQMAYRF | [Tarke et al. 2021](https://www.sciencedirect.com/science/article/pii/S266637912100015X) |
|  | FDNPVLPFNDGVYF | [Tarke et al. 2021](https://www.sciencedirect.com/science/article/pii/S266637912100015X) |
|  | FPQSAPHGVVF | [Tarke et al. 2021](https://www.sciencedirect.com/science/article/pii/S266637912100015X) |
|  | FVSNGTHWF | [Tarke et al. 2021](https://www.sciencedirect.com/science/article/pii/S266637912100015X) |
|  | IPFAMQMAY | [Tarke et al. 2021](https://www.sciencedirect.com/science/article/pii/S266637912100015X) |
|  | LGAENSVAY | [Tarke et al. 2021](https://www.sciencedirect.com/science/article/pii/S266637912100015X) |
|  | LPFNDGVYF | [Tarke et al. 2021](https://www.sciencedirect.com/science/article/pii/S266637912100015X) |
|  | LPIGINITRF | [Tarke et al. 2021](https://www.sciencedirect.com/science/article/pii/S266637912100015X) |
|  | LPPAYTNSF | [Tarke et al. 2021](https://www.sciencedirect.com/science/article/pii/S266637912100015X) |
|  | LPPLLTDEM | [Tarke et al. 2021](https://www.sciencedirect.com/science/article/pii/S266637912100015X) |
|  | LTDEMIAQY | [Tarke et al. 2021](https://www.sciencedirect.com/science/article/pii/S266637912100015X) |
|  | NATRFASVY | [Tarke et al. 2021](https://www.sciencedirect.com/science/article/pii/S266637912100015X) |
|  | QIPFAMQMAY | [Tarke et al. 2021](https://www.sciencedirect.com/science/article/pii/S266637912100015X) |
|  | QPTESIVRF | [Tarke et al. 2021](https://www.sciencedirect.com/science/article/pii/S266637912100015X) |
|  | SANNCTFEY | [Tarke et al. 2021](https://www.sciencedirect.com/science/article/pii/S266637912100015X) |
|  | TSNQVAVLY | [Tarke et al. 2021](https://www.sciencedirect.com/science/article/pii/S266637912100015X) |
|  | VASQSIIAY | [Tarke et al. 2021](https://www.sciencedirect.com/science/article/pii/S266637912100015X) |
|  | WTAGAAAYY | [Tarke et al. 2021](https://www.sciencedirect.com/science/article/pii/S266637912100015X) |

^a^BNT162b2 vaccine-derived immunogenic epitopes identified in this study.
